# The Gut Microbiota: Emerging Evidence in Autoimmune and Inflammatory Diseases

**DOI:** 10.34133/research.1097

**Published:** 2026-02-04

**Authors:** Liuting Zeng, Qianyue Yang, Yong Luo, Yanfang Luo, Lingyun Sun

**Affiliations:** ^1^ Department of Rheumatology and Immunology, Nanjing Drum Tower Hospital, Chinese Academy of Medical Sciences & Peking Union Medical College, Nanjing, Jiangsu, China.; ^2^ Department of Rheumatology and Immunology, Nanjing Drum Tower Hospital Clinical College of Nanjing Medical University, Nanjing, Jiangsu, China.; ^3^ Department of Rheumatology and Immunology, Nanjing Drum Tower Hospital, Nanjing University of Chinese Medicine, Nanjing, Jiangsu, China.; ^4^ Department of Nephrology, The Central Hospital of Shaoyang, Affiliated with the University of South China, Shaoyang, Hunan, China.; ^5^ State Key Laboratory of Pharmaceutical Biotechnology, Nanjing University, Nanjing, Jiangsu, China.

## Abstract

Autoimmune diseases (AIDs) are a group of immune-related disorders primarily affecting joints and surrounding tissues, often marked by chronic inflammation and autoimmune activation. Common types include systemic lupus erythematosus, rheumatoid arthritis, multiple sclerosis, autoimmune cardiovascular diseases, and skin conditions. While their pathogenesis is unclear, recent studies suggest that abnormal gut microbiota may contribute. Previous research has shown that various patients with rheumatic disease exhibit altered gut microbiota, characterized by decreased microbial diversity, overall compositional changes, and microbiota-mediated functional alterations. Bacterial species closely associated with AIDs include *Prevotella copri*, *Ruminococcus gnavus*, and *Ligilactobacillus salivarius*. Dysregulated gut microbiota activates host immune responses through multiple mechanisms, including compromised intestinal barrier, systemic translocation, molecular mimicry of self-antigen epitopes, and changes in microbiota-derived metabolites, thereby substantially contributing to the development and progression of AIDs. Microbial metabolites, including short-chain fatty acids, tryptophan metabolites, and bile acid metabolites, are actively involved in driving disease progression. In addition, the therapeutic outcomes and adverse effects of immunotherapeutic agents can be modulated by gut microbiota through their impact on drug biotransformation processes. Clinically, analyzing gut microbiota characteristics can aid in disease diagnosis and prognosis prediction. Therapeutic strategies such as fecal microbiota transplantation, probiotics, prebiotics, and the Mediterranean diet may become effective measures for managing AIDs. This article reviews recent research progress, future directions, and the potential of microbiota-based interventions in treating AIDs.

## Introduction

The diverse microbial communities within the human gut collectively maintain the balance of gut microbiota and play crucial roles in regulating functions such as digestion, metabolism, immunity, and energy conversion [1]. In recent years, an increasing body of research has reported on the relationship between gut microbiota and immune therapy [2]. The interactions between gut microbiota and the human immune system represent a complex dynamic equilibrium process involving aspects such as immune cell development, functional regulation, and pathogen defense [3]. These interactions are vital for maintaining human health, and when the balance between gut microbiota and the human immune system is disrupted, diseases can ensue [3]. Autoimmune diseases (AIDs) are a category primarily affecting joints and surrounding soft tissues, often manifested by sustained chronic inflammation and self-immune activation [4]. Currently incurable, these conditions are mainly managed through long-term use of potent steroids and immunosuppressants to control symptoms [5]. The mechanistic underpinnings and causal pathways of autoimmune disorders remain partially unveiled. While hereditary predispositions and external exposures, including tobacco use, environmental toxins, microbial infections, and antigenic cross-reactivity, have been implicated in the onset of autoimmune conditions [6], emerging research highlights that lifestyle-associated alterations in intestinal microbial communities may constitute a pivotal driver of the escalating prevalence of these disorders [7,8]. The human gastrointestinal tract harbors a wide range of microorganisms, including bacteria, fungi, and viruses, which interact with and regulate the human immune system [9]. Recent studies have shown that a multitude of genetic and environmental factors can enhance susceptibility to such diseases [10]. Epidemiological research indicates a rising trend in the incidence of common AIDs, including inflammatory bowel disease, systemic lupus erythematosus (SLE), and inflammatory arthritis over recent years [11]. Studies suggest that this increase could be related to reduced exposure to a variety of exogenous microorganisms and heightened sensitivity of the immune system due to modernized lifestyles [12,13].

The rapid development of DNA high-throughput sequencing technologies has shifted microbiota research beyond inefficient and stringent in vitro bacterial cultures, enabling deeper insights through multiomics approaches such as metagenomics. The gut microbiome, with a gene pool approximately 150 times larger than the human genome, exhibits unique metabolic activities [14,15]. As the largest immune organ in the body, the gut harbors abundant immune cells and molecules, particularly in the lamina propria and gut-associated lymphoid tissue (GALT). The establishment and homeostasis of gut microbiota are closely intertwined with the development and function of the host immune system, collectively maintaining resistance to pathogens and appropriate immune responses to antigens [16,17]. Studies suggest that gut microbiota is a critical prerequisite for autoimmune responses. Clinical cohort studies worldwide, including our prior research [18,19], using next-generation sequencing and multiomics methods, have revealed significant dysbiosis in patients with AIDs (e.g., lupus, autoimmune neurological disorders, inflammatory arthritis, autoimmune cardiovascular diseases, and autoimmune enteritis). These changes include reduced microbial diversity, overgrowth of harmful bacteria, increased gut barrier permeability leading to bacterial translocation, and molecular mimicry of self-antigens by bacterial peptides, all contributing to autoimmune and inflammatory diseases. In addition, gut microbiota in AIDs exhibits distinct metabolic profiles, such as altered levels of short-chain fatty acids (SCFAs), tryptophan metabolites, and bile acid metabolites, which enter the bloodstream and drive immune dysregulation in multiple organs [20]. This review details changes in gut microbiota composition and function in various autoimmune and inflammatory diseases, explores the mechanisms by which microbiota and their metabolites regulate these conditions, and discusses the potential of microbiota-related diagnostic markers and therapeutic targets, as well as microbiota-based interventions such as fecal microbiota transplantation (FMT), probiotics, prebiotics, and Mediterranean diets in the diagnosis and treatment of autoimmune and inflammatory diseases.

## Aberrant Gut Microbiota in AIDs and Potential Pathogenic Mechanisms

Metagenomic sequencing studies confirm distinct gut microbiota composition between patients with AID and healthy controls, reflecting microbial ecological imbalance [18–20]—a pattern also observed in irritable bowel syndrome and celiac disease. Beyond taxonomic changes (species richness/abundance), dysbiosis-driven alterations in microbial metabolic functions (biosynthetic/degradation pathway abnormalities) disrupt gut ecology and induce pathological damage. The gut microbiota–host immunity cross-talk underpins AIDs development, with immune dysregulation mediated by microbial translocation, molecular mimicry, dysbiosis-related metabolite (e.g., SCFAs) disturbances, epitope spreading, and bystander activation.

### Gut microbiota abnormalities

Microbial imbalance is a shared feature across AIDs, with disease-specific taxonomic shifts: (a) SLE: 16*S* ribosomal RNA sequencing in Spanish/Chinese cohorts shows reduced *Firmicutes*/*Bacteroidetes* ratio [21,22] (*Firmicutes* include butyrate-producing symbionts, key for immunomodulation). Shotgun sequencing identifies enriched *Bacteroides fragilis* and *Clostridium leptum* in SLE guts (levels decrease posttreatment) [23]; oral bacteria (*Atopobium rimae*, *Shuttleworthia satelles*, and *Actinomyces massiliensis*) translocate to the gut [23]. Active *Ruminococcus gnavus* (*Veillonellaceae*) is enriched in lupus nephritis (LN), correlating with disease activity [24]. (b) Rheumatoid arthritis (RA): Gut microbiota shows elevated *Prevotella* spp. and reduced *Bacteroides* spp. [25], alongside increased *L. salivarius* and decreased *Bifidobacterium bifidum* [26]. Periodontopathic bacteria (*Porphyromonas gingivalis* and *Aggregatibacter actinomycetemcomitans*) induce anticitrullinated protein antibodies (ACPAs), driving RA development. (c) Primary biliary cholangitis (PBC): Decreased *Bacteroides*/*Prevotella* and increased *Haemophilus*/*Veillonella*/*Lactobacillus*/*Streptococcus*; these changes partially reverse with ursodeoxycholic acid (UDCA) therapy [27]. (d) Ankylosing spondylitis (AS): Early studies note increased *Ruminococcaceae*, *Lachnospiraceae*, *Erysipelotrichaceae*, *Faecalibacterium*, and *Bacteroidaceae* and reduced *Veillonellaceae* and *Prevotellaceae*; species-level analyses confirm enriched *Prevotella melaninogenica*, *Prevotella copri*, and *Prevotella* sp. C561, as well as active *Ruminococcus* (correlates with disease activity) [28,29]. (e) Sjögren’s syndrome (SS): Dysbiosis features enriched mucin-degrading pathogens and reduced butyrate-producing *Bacteroidetes* and *Firmicutes*, associating with dryness symptoms and disease activity [30].

Shared microbial patterns are observed across AIDs, despite differences in sequencing methods and study cohorts. These include the following: (a) *Prevotella* expansion and reduced butyrate-producing *Faecalibacterium prausnitzii* in RA [31], SS [31], and AS [29]; (b) enriched active *Ruminococcus* in SLE [24], inflammatory bowel disease (IBD) [32], and spondyloarthropathy (SpA) [33]; (c) periodontopathic bacteria correlate with anti-double-stranded DNA (dsDNA) antibody titers/complement activity in RA [34], beyond their role in SLE [35], suggesting shared microbial triggers and analogous immune dysregulation.

Notably, phylum-level changes may contradict class/order/species-level shifts. Advances in sequencing and mechanistic research will deepen understanding of gut microbiota taxonomy and function in AIDs.

### Potential pathogenic mechanisms

#### Gut microbiota translocation and molecular mimicry

In SLE, *Enterococcus gallinarum* and *Limosilactobacillus reuteri* translocate to the liver/lymphoid tissues (confirmed by tissue culture/in situ hybridization) and activate plasmacytoid dendritic cells (DCs) to promote type I interferon (IFN-I) production [36,37]. *Roseburia intestinalis* and *Bacteroides thetaiotaomicron* induce autoantibodies (anti-β_2_-glycoprotein I and anti-Ro60) via nonorthologous sequences [38,39].

In SS, *Escherichia coli* (carrying von Willebrand factor A) and oral *C. ochracea* activate Ro60-specific T cells, suggesting molecular mimicry [40]. In AS, *Klebsiella pneumoniae* interacts with human leukocyte antigen B27 (HLA-B27) via antigenic mimicry to drive disease [41]. In addition, peptides from gut-enriched bacteria induce proinflammatory factor release from T cells of patients with AID (recall-response-like), supporting microbial involvement in autoimmunity via mimicry [23,41].

#### SCFA imbalance

SCFAs, including acetate, propionate, and butyrate—microbial metabolites derived from dietary fiber—regulate immunity through mechanisms such as modulating oxidative phosphorylation and glycolipid metabolism, inhibiting histone deacetylases (HDACs) (thereby controlling gene expression), and suppressing the nuclear factor κB (NF-κB) pathway to exert anti-inflammatory effects. Butyrate, the key SCFA, promotes immune tolerance by inducing tolerogenic DCs/regulatory T cells (Treg cells), M2 macrophage polarization, and interleukin-10 (IL-10) secretion; it also maintains intestinal barrier integrity via epithelial repair and mucus synthesis [42,43].

Dysregulation of SCFAs is increasingly linked to AIDs. Patients with RA have reduced SCFAs, and butyrate supplementation has been shown to alleviate disease severity in murine models of arthritis [44]. Similarly, in lupus-prone mice, butyrate administration attenuates cutaneous and renal inflammation [45]. Moreover, antibiotic use—which depletes SCFA-producing commensals and consequently lowers SCFA concentrations is associated with an elevated risk of AIDs, further underscoring the protective role of these microbial metabolites in immune homeostasis.

#### Bystander activation and epitope expansion

Bacterial lipopolysaccharides (LPS) activate DCs to release proinflammatory factors, driving T cell “bystander activation” (antigen/T cell receptor [TCR]-independent) via inflammatory signals/Toll-like receptor (TLR) stimulation.

As inflammation progresses, exposed new antigen epitopes trigger cross-reactions between self-antigens and antimicrobial antibodies (epitope expansion): In RA, *P. gingivalis* expands immune responses from noncitrullinated to citrullinated peptides [46]; in SLE, *E. gallinarum* translocation induces anti-RNA/anti-dsDNA antibodies and amplifies autoimmunity via anti-β_2_-glycoprotein I production [36,47].

Notably, microbial roles are strain specific: *Lactobacillaceae* are generally beneficial (e.g., certain *Lactobacillus* strains reduce SLE-like disease in mice via Treg/T helper 17 (Th17) cell balance and antioxidant effects [48–50]), but patients with SLE have enriched *Lactobacillus* (correlating with disease activity), and *L. reuteri* exacerbates murine lupus via plasmacytoid DC activation/IFN-I release [51], highlighting context-dependent microbial–immune interactions.

**Fig. 1.**
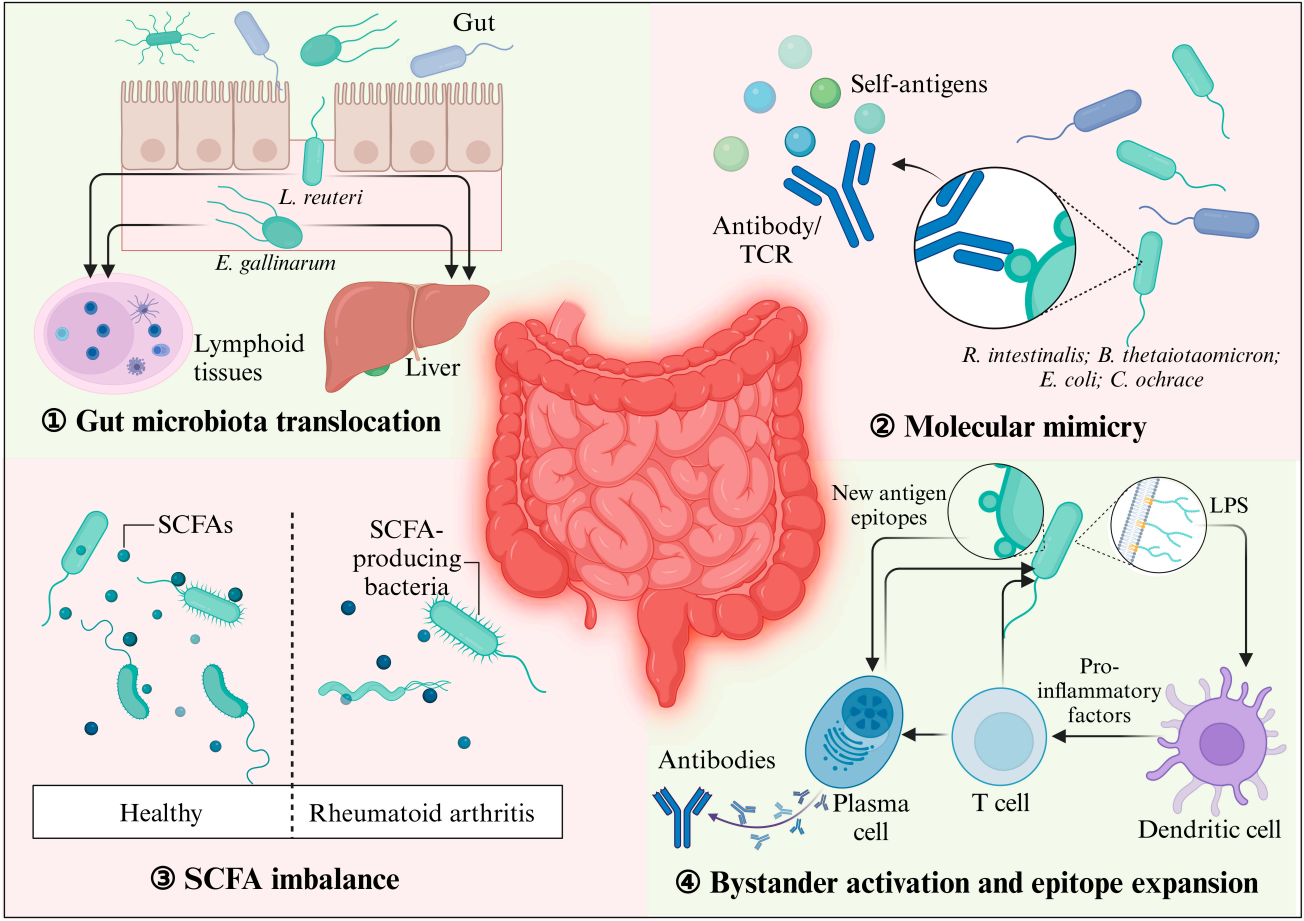
The potential pathogenic mechanisms of the gut microbiota on AIDs. The gut microbiota influences AIDs through multiple mechanisms. Bacteria such as *E. gallinarum* and *L. reuteri* can migrate to lymphoid tissues and the liver, triggering immune responses and autoantibody production. Some microbial antigens resemble host self-antigens, causing cross-reactivity and autoimmune reactions. (SCFAs, produced by gut bacteria, regulate immunity but are reduced by antibiotics, affecting conditions such as RA). Inflammatory environments also promote T cell bystander activation, leading to autoantibody production. This figure was created using BioRender.com.

In conclusion, the intestinal microbial ecosystem exhibits remarkable complexity, and in-depth understanding is required in terms of how gut commensals change functionally alongside the host’s status, disease progression, and environmental factors to further explore the key immune pathways involving the gut microbiota so as to promote microbiota-based intervention therapies (Fig. 1).

Notably, the gut microbiota and host immune regulation interact dynamically, with microbial metabolites such as SCFAs, tryptophan derivatives, and bile acids playing key roles in immune cell function and AID progression. However, gaps remain in understanding the full scope of these metabolites and their mechanisms, including challenges in distinguishing host-derived from microbiota-derived metabolites and quantifying their physiological levels. Advances in metagenomics and metabolomics offer powerful tools to further explore microbial metabolite dynamics; while clinical applications are still emerging, research into microbial metabolite–immune interactions will clarify disease mechanisms and drive targeted therapies, potentially transforming AID management through personalized, microbiota-based interventions.

## Gut Microbiota and AIDs

### The role of gut microbiota dysbiosis in the pathogenesis of SLE

SLE is a heterogeneous systemic AID characterized by multisystem/organ involvement, recurrent flares, abundant autoantibodies, and potential irreversible organ damage without timely intervention [52]. Its etiology involves genetics, sex hormones, environmental triggers, and dysregulation of innate/adaptive immunity—including excessive IFN-I secretion, aberrant B cell activation (e.g., anti-dsDNA antibodies), T cell subset imbalance (impaired Treg cell function and excessive Th17 cell proliferation), and neutrophil extracellular trap (NET)-driven inflammation [53–55]. Patient-specific dominant pathogenic pathways (e.g., B cell hyperactivity versus IFN-I activation) support targeted therapies [56], while cutting-edge technologies have identified pathogenic immune subsets (age-associated B cells, follicular helper T [Tfh] cells, IFN-γ-producing CD8^+^ T cells, and antibody-secreting cells) linked to disease activity [57–60]. Therapeutic strategies are individualized: Mild cases may respond to hydroxychloroquine plus low-dose glucocorticoids, while severe visceral involvement or high disease activity (per 2023 European Alliance of Associations for Rheumatology guidelines) warrants early biologics (e.g., belimumab and anifrolumab) [61,62]. Emerging evidence suggests that genetic susceptibility-related gut microbiota instability and pathobionts contribute to SLE pathogenesis [63].

Compared to healthy individuals, patients with SLE exhibit reduced gut microbial richness and diversity [64–68]. Key findings include immunostimulation by Ro60 homolog-expressing bacteria (driving SLE-related autoimmunity) [64] and conflicting alterations in the *Firmicutes*/*Bacteroidetes* ratio, highlighting microbial complexity in SLE [67,69,70]. Hevia et al. [71] reported a reduced *Firmicutes*/*Bacteroidetes* ratio in patients with SLE, implying therapeutic potential of microbial modulation. Zhang et al. [72] linked microbiota structural changes to peripheral blood lymphocyte counts and SLE disease activity, suggesting regulatory roles in disease onset/progression. Anti-dsDNA antibodies, a core marker of SLE activity [73], are closely associated with microbial perturbations [74].

**Fig. 2.**
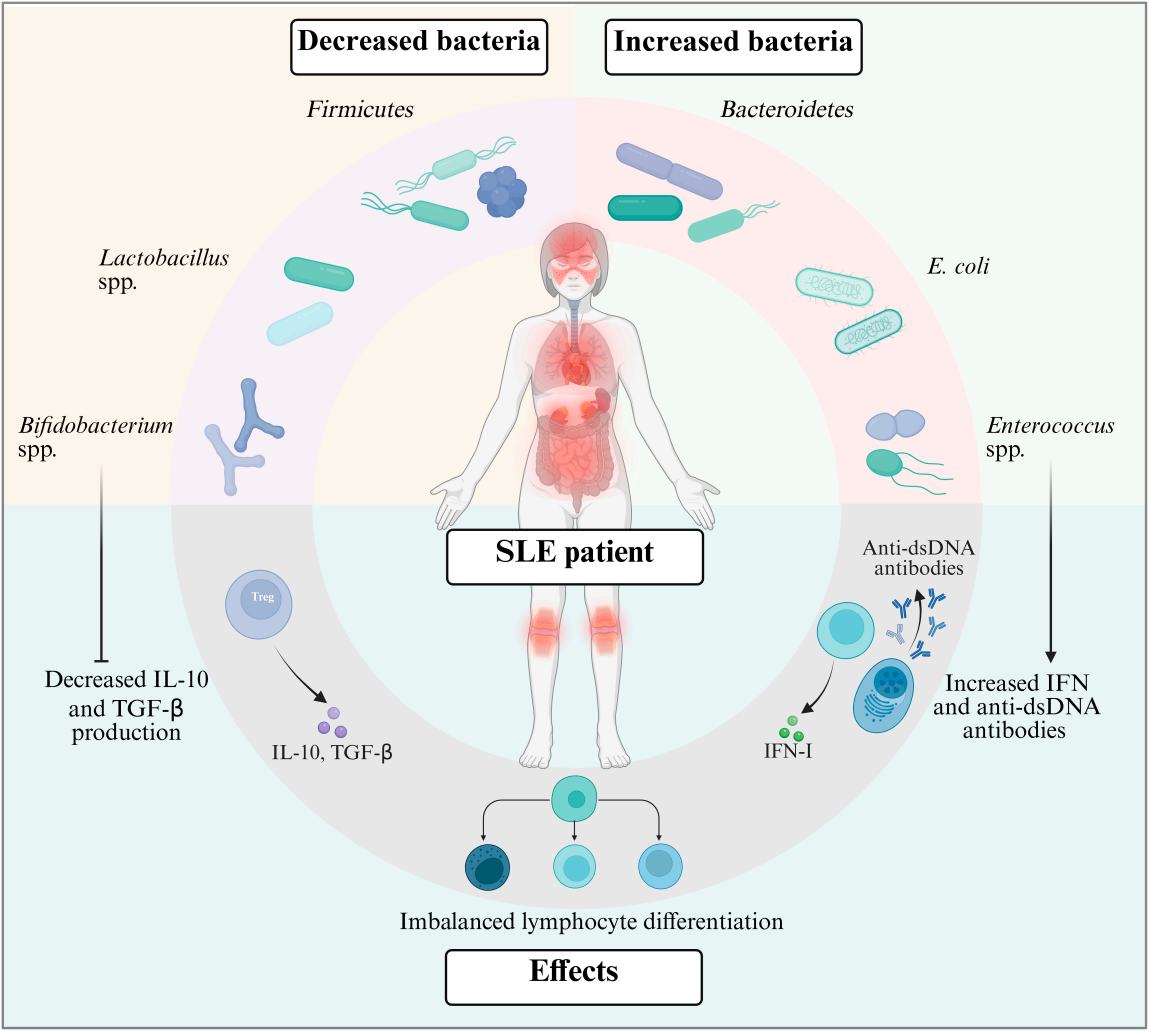
The role of the gut microbiota in the pathogenesis of SLE. Various bacterial strains are decreased in the intestines of patients with SLE, including *Firmicutes*, *Lactobacillus* spp*.*, and *Bifidobacterium* spp., while others, including *Bacteroidetes*, *E. coli*, and *Enterococcus* spp., are found to be elevated. Since *Bifidobacterium* aids in the Treg cell production of IL-10 and TGF-β, patients with SLE often present with decreased IL-10 and TGF-β levels. The enrichment of *Enterococcus*, on the other hand, increases IFN and anti-dsDNA antibodies. Meanwhile, gut microbiota dysbiosis in patients with SLE also results in the imbalanced differentiation of lymphocytes. This figure was created using BioRender.com.

The gut microbiota is a critical target for SLE research, with multifaceted mechanisms linking dysbiosis to disease [74]. Pathogenic mechanisms include translocation of *Enterococcus* to systemic tissues (driving IFN expression and autoantibody production) [7,75,76], reduced beneficial bacteria (*Lactobacillus* and *Bifidobacterium*) and increased pathogens (*E. coli* and *Enterococcus*) in SLE [77], and SLE microbiota-induced elevation of anti-dsDNA antibodies and immune activation in germ-free (GF) mice [78]. Kim et al. [79] further demonstrated that *Enterococcus* and *L. reuteri* induce IFN-I and anti-dsDNA antibodies in patients with SLE. Gut microbiota also modulates SLE-related immune balance (Th17/Treg cell and transforming growth factor-β [TGF-β]/IL-17) via regulating cytokines (tumor necrosis factor-α [TNF-α], IL-6, and IL-10) and B cell function [80–82]. Collectively, gut microbiota dysbiosis disrupts immune homeostasis to drive SLE initiation and progression, although causal relationships and detailed mechanisms remain to be elucidated. In comparison to healthy individuals, patients with SLE exhibit a decreased relative abundance of *Firmicutes*, increased *Bacteroidetes*, elevated *Lactobacilli*, and an association between increased *A. actinomycetemcomitans* and disease activity as well as the incidence of LN (Fig. 2 and Box 1).

Box 1. Gut microbiota and microbial metabolites in SLE pathogenesis

SCFAs

• Dysbiosis feature: Reduced SCFA-producing bacteria (e.g., *Lactobacillus* and *Bifidobacterium*)
• Metabolic shift: 65% of altered serum metabolites are lipid-related, indicating lipid metabolism dysregulation
• Therapeutic potential: Resistant starch/SCFA supplementation suppresses *L. reuteri* and mitigates lupus phenotypes in mice

Tryptophan metabolites

• Aryl hydrocarbon receptor (AhR) modulation: Decreased circulating AhR agonists (indole derivatives) correlate with disease activity
• Barrier impact: Certain indoles enhance gut barrier integrity; others may promote inflammation
• Clinical correlation: Altered tryptophan metabolism linked to SLE disease progression

Bile acids

• Signature change: Fecal primary bile acids increase (cholic/glycocholic/taurocholic acids), while secondary bile acids decrease
• Receptor dysfunction: Farnesoid X receptor (FXR) down-regulation exacerbates liver damage; activation alleviates inflammation
• Therapeutic target: UDCA modulates bile acid circulation and microbiota

#### LN and gut microbiota

LN, a severe SLE complication, affects over 50% of patients with SLE and accounts for ~70% of secondary glomerular diseases [83]. LN progression alters gastrointestinal structure, barrier function, and microbial populations, reducing gut microbiota tolerance. This imbalance, exacerbated by gut-barrier-disrupting environmental factors, triggers systemic inflammation via neutrophil infiltration, proinflammatory macrophage differentiation, and Th1/Th17 cell polarization [84]. Malnutrition further disrupts TLR expression and Th17/Treg cell balance [85], while gut microbiota composition/metabolites regulate antibody production, B cell activity, and Th17/Treg cell homeostasis [86]. For instance, *Lactobacillus* species promote Treg-cell-mediated IL-10 production and suppress Th17 cells via TLR2 signaling (polysaccharide-A-dependent) [87,88], and lupus-prone mice exhibit disease-severity-correlated reductions in *Lactobacillus* and increases in *Spirochaetes* [89].

The healthy gut microbiota forms a mucosal barrier against antigen invasion and coordinates with innate/adaptive immunity to minimize microbe–epithelial contact, underscoring its role in LN pathogenesis. LN-associated immune dysregulation impairs this barrier, while gut microbiota dysbiosis is linked to SLE progression. In animal models, studies using female lupus-prone mice have revealed significant alterations in the gut microbiota, characterized by reduced *Lactobacilli*, increased abundance of *Lachnospiraceae*, and elevated overall diversity. Furthermore, the enrichment of *Clostridiaceae*/*Lachnospiraceae* during SLE progression suggests that supplementation with probiotic *Lactobacilli* or retinoic acid may help alleviate inflammation [90]. A murine MRL/lpr model study demonstrated that a combination of 5 *Lactobacillus* strains (*Ligilactobacillus oris*, *Lacticaseibacillus rhamnosus*, *L. reuteri*, *Lacticaseibacillus johnsonii*, and *Lacticaseibacillus gasseri*) reverses leaky gut via enhanced *Lactobacillus* colonization. These strains reduce gut IL-6, enhance IL-10 (promoting anti-inflammatory milieu), systemically elevate IL-10, and reduce renal immunoglobulin G2a (IgG2a) deposits, and shift renal Treg/Th17 balance toward Treg cells. Notably, these benefits are observed in female and castrated male mice but not intact males, indicating sex-hormone-dependent modulation of LN by gut microbiota [91]. Limited human studies exist on gut dysbiosis in LN, and further research is needed to clarify its pathogenic mechanisms and clinical relevance.

In summary, SLE genetic predisposition correlates with gut microbiota instability, enabling pathogenic strains to expand and compromise intestinal barrier integrity. Increased gut permeability exposes the immune system to bacterial components and proinflammatory metabolites, perpetuating chronic inflammation and displacing commensal bacteria. Antibodies against microbial antigens may cross-react with host autoantigens, forming immune complexes and activating T-cell-mediated pathways, highlighting the complex interplay between microbial dysbiosis, immune dysregulation, and LN pathogenesis.

#### Targeting gut microbiota for intervention in SLE

Various strategies targeting gut microbiota dysbiosis in SLE hold potential for restoring intestinal homeostasis and improving clinical outcomes, although the direct cause–effect relationship between microbiota modulation and therapeutic efficacy remains to be fully elucidated. Glucocorticoid therapy has been shown to influence the gut microbiota of patients with SLE: Dexamethasone treatment significantly increases gut bacterial diversity and reduces *Lactobacillus* species associated with SLE severity [92], while glucocorticoid use can also elevate probiotic taxa (*Bifidobacterium* and *Lactobacillus*) and restore the healthy *Firmicutes*/*Bacteroidetes* ratio [93], suggesting potential microbiota-normalizing effects. It should be noted that these observed microbiota changes could be either a contributing factor to therapeutic responses or a secondary effect of glucocorticoid-induced systemic physiological alterations.

Probiotics (notably *Lactobacillus* and *Bifidobacterium*) and prebiotics are another key approach, controlling inflammation, reducing autoantibody production, and mitigating SLE severity [94,95]. For example, *Bifidobacterium* maintains Treg/Th17/Th1 cell balance by inhibiting excessive CD4^+^ T cell activation in patients with SLE [66]. FMT is highly effective for microbiota restoration. Huang et al. [96] conducted a 12-week single-arm trial: 20 active patients with SLE received 30 oral FMT capsules (from 7 healthy donors) plus standard treatment. Forty-two point 12% (42.12%) achieved the primary end point (SRI-4 [SLE responder index-4]), with significant reductions in urinary protein, serum anti-dsDNA titers/antibody levels, and systemic lupus erythematosus disease activity index scores. FMT enriched SCFA-producing bacterial taxa, reduced inflammation-related taxa, increased gut SCFA synthesis, and lowered peripheral blood IL-6 levels and CD4^+^ memory/naive ratios, with no severe adverse events or deaths. A recent study [97] confirmed FMT reshapes the gut microbiome by modulating B cell differentiation and reducing autoantibody production, ameliorating SLE progression. The post-FMT increase in *L. johnsonii* and its mediated purine metabolic pathway were identified as key contributors, highlighting the value of microbiome-targeted therapies.

In summary, as research progresses, it is increasingly hypothesized that because of genetic susceptibility, patients with SLE may be at heightened risk for instability and fluctuations in their gut microbiota. Microbial strains harboring complementary genetic elements may gain a competitive growth advantage within the intestinal niche, leading to increased microbial abundance and potentially enabling the overgrowth of specific pathogenic taxa. Certain pathogenic species, particularly those colonizing the ileal mucosal surface, can directly compromise intestinal barrier integrity, thereby enhancing gut permeability and consequently increasing host exposure to bacteria themselves or their proinflammatory products. This increase in intestinal permeability may initiate a feed-forward loop, further amplifying systemic exposure to translocated microbes, microbial DNA, antigens, and metabolites, thus perpetuating chronic inflammation. These processes, in turn, may reshape the local intestinal microenvironment to favor the expansion of inflammation-adapted strains over less competitive commensals. As antibodies against microbial antigens are generated, cross-reactive antibodies targeting host self-antigens may also emerge. The formation of immune complexes and/or stimulation of T-cell-mediated pathways may further accelerate disease progression. In the future, novel therapeutic strategies—including dietary modifications, administration of probiotics, or pathogen-specific immunotherapies—may help restore intestinal barrier integrity and rebalance the gut microbial community. Such interventions could promote systemic immune homeostasis, reverse metabolomic alterations and inflammatory milieus associated with AID, and ultimately ameliorate clinical outcomes in patients.

### Gut microbiota in RA

RA is a chronic systemic AID primarily targeting joint synovium, cartilage, and bone—persistent synovitis leads to structural destruction, deformity, disability, and extra-articular involvement (skin, vasculature, eyes, and respiratory system) [98]. Pathogenically, RA arises from genetic–environmental interactions [98], initiating with sustained cellular activation and progressing to organ-targeted autoimmunity [99]. Fibroblast-like synoviocytes (FLSs) are pivotal in pathogenesis [100–102], with disease progression divided into 3 phases: T-cell-amplified nonspecific synovial inflammation, chronic inflammation, and tissue destruction mediated by IL-1, IL-6, and TNF-α [103–106]. RA synovitis features accumulation of innate/adaptive immune cells (T cells, DCs, B cells, macrophages, and osteoclasts), whose proinflammatory/bone-destructive mediators drive synovial hyperplasia, angiogenesis, and joint damage [107–110]. Genetic/environmental triggers activate innate immunity via DC stimulation, recruiting T cells that further induce B cells/macrophages/synoviocytes to secrete IL-1β, IL-6, TNF-α, and matrix metalloproteinases [111,112], ultimately causing cartilage/bone destruction. Clinically, inflammatory-cytokine-targeted therapies are first line [113].

A key pathogenic hypothesis proposes RA initiation at mucosal sites, driven by mucosal immune system–dysbiotic microbiota cross-talk, prior to synovial involvement [114]. Microbial alterations in the lungs, oral cavity, and gut of patients with preclinical/established RA support mucosal dysbiosis in disease onset/progression, although distinguishing primary etiology from secondary inflammation-induced changes remains unresolved [115]. Murine model data and preclinical studies confirm that microbiota alterations precede clinical manifestations [116], while RA therapeutics correlate with gut microbiota shifts, highlighting the potential of microbiome/intestinal barrier modulation for RA prevention/treatment [117]. Gut microbiota dysbiosis (environmental/host-factor-driven) disrupts commensal-pathogen balance, impairing mucosal immune tolerance and triggering excessive inflammation [118]. Commensals support mucosal immune maturation, while pathogens induce proinflammatory cytokine (IL-1β, IL-6, TNF-α, and IL-17) imbalance—a core RA driver [118]. Genome-wide studies show RA autoantibodies precede clinical symptoms, with the gut as a key early inflammatory niche [119]. Altered gut microbiota abundance is an RA hallmark [120], strongly supporting the mucosal origin hypothesis.

Gut microbiota composition/function is altered in RA [121], but longitudinal predisease studies are needed to confirm causality. Alpizar-Rodriguez et al. [122] identified *P. copri* (a *Bacteroidetes* commensal) enrichment in preclinical RA, which drives Th17 cell responses in vitro. Larsen et al. [123] observed high *Prevotella* levels in early untreated RA, suggesting immunoregulatory roles. Patients with RA also exhibit increased *Collinsella aerofaciens*/*Eggerthella lenta* and decreased *Faecalibacterium* [124]—changes linked to intestinal barrier impairment and elevated inflammation risk. Notably, gut *Lactobacillus* abundance is significantly higher in patients with RA than healthy controls [125], indicating strain-specific pathogenicity (some *Lactobacillus* may promote arthritis). Collectively, clinical evidence confirms that microbial dysbiosis precedes RA onset and exerts immunomodulatory effects and that FMT is a promising therapeutic strategy.

Adjuvant-induced arthritis (AIA) and collagen-induced arthritis (CIA) are canonical RA models with contrasting microbiota effects: GF rats with AIA show exacerbated inflammation [126,127], while antibiotic-pretreated CIA mice exhibit reduced disease severity, lower inflammatory cytokines, and antibodies against type II collagen (anti-CII) antibodies [127,128]. Gut dysbiosis and intestinal inflammation precede CIA onset and persist throughout disease, with intestinal inflammation alleviation improving outcomes [127,128]. GF or antibiotic-treated K/BxN and *IL1rn*^−/−^ mice show attenuated arthritis, but reintroducing segmented filamentous bacteria (SFB) in K/BxN mice induces lamina propria Th17 cell differentiation and autoantibody production via cecal-starch-like antigen-A-targeted IgA, triggering recurrence [129,130]. *Il17ra*^−/−^ mice (IL-17Ra-deficient) have reduced gut microbiota diversity, increased *Helicobacter*, and decreased *Ruminococcus*/*Prevotella* [131]—these changes induce TLR4 activation and Th17 cell differentiation, with effects transmissible to wild-type mice via FMT [132]. Critically, transferring *Prevotella* from patients with RA into SKG mice up-regulates IL-6/IL-23, promotes Th17 cell polarization, and induces RA [133]. These findings confirm gut microbiota drives arthritis via Th17 cell differentiation in murine models, although model-specific mechanism differences warrant further investigation into strain-specific pathogenicity.

#### Impairment of intestinal barrier integrity in patients with RA or animal models

The intestinal mucosal immune system—comprising the mucus layer, epithelial cells, and lamina propria—functions as a critical immune surveillance network. Intestinal epithelial cells form a selective barrier via tight junctions (TJs), adherens junctions, and desmosomes [134,135], regulating permeability to isolate the intestinal lumen from systemic circulation, prevent translocation of gut microbiota/their harmful metabolites, and facilitate nutrient absorption [136]. The mucus layer (predominantly MUC2 mucin, IgA, and enzymes [137,138]) protects epithelial cells from mechanical/chemical/biological damage [139]; GF mice have a thin colonic mucus layer that thickens upon exposure to bacterial LPS or peptidoglycan [140]. The lamina propria harbors T/B cells: Naive CD4^+^ T cells differentiate into Th/Treg cells, while Peyer’s patch-activated B cells mature into IgA-secreting plasma cells, fulfilling immune surveillance roles [141]. Pothoven et al. [142] proposed the “barrier hypothesis” for RA pathogenesis: Intestinal mucosal barrier damage (“leaky gut”) allows bacterial translocation, activating mucosal immune cells and triggering systemic immune responses and joint inflammation—the “gut–joint axis” hypothesis. Gut lamina propria Th17 cells secrete IL-17A, IL-17F, and IL-22, inducing epithelial cells to produce antimicrobial peptides and TJ proteins to maintain barrier integrity [143,144]. Excessive Th17 cell responses are closely linked to RA [145,146], and CIA mice exhibit compromised intestinal barrier function prior to arthritis onset, accompanied by elevated Th17-cell-related cytokines (IL-17A, IL-22, and IL-23) [147]. Antibodies targeting Th17-cell-related cytokines are already used in RA therapy [148]. Zonulin is a key regulator of intestinal TJs, reducing barrier function by degrading zona occludens-1 (ZO1) and occludin [149]. Elevated zonulin expression in autoimmune mice and patients correlates with barrier leakage, dysbiosis, and inflammation; zonulin receptor antagonists improve intestinal barrier indices and suppress arthritis in mice [150]. Bacterial activity influences zonulin expression, suggesting a link between gut dysbiosis and intestinal barrier impairment in RA [150]. While the “mucosal barrier origin hypothesis” faces challenges in proving barrier impairment as the earliest event in RA, targeted repair of damaged mucosal barriers yields therapeutic benefits—highlighting barrier damage as a driver of RA progression and a potential treatment target.

#### Mechanism of intestinal mucosal immune regulation in RA

RA is characterized by autoantibodies such as ACPAs and rheumatoid factor (RF) [151,152]. In high-risk and healthy individuals, IgA-type ACPA and RF are often linked to the intestinal mucosa. IgA plasma cells in Peyer’s patches produce mucosal IgA, which neutralizes pathogens and blocks their translocation across the epithelium. When barrier integrity is compromised, translocated bacteria are recognized by DCs, triggering inflammation and Treg cell responses [153]. Although IgA typically serves protective roles, elevated levels within immune complexes can damage joints [154], suggesting that intestinal mucosal immunity contributes to early RA pathogenesis. Notably, co-occurrence of ACPA and RF synergistically accelerates joint destruction [155].

Multiple immune cell subsets further link gut immunity to RA.

1. Innate lymphoid cells (ILCs): ILCs mirror Th cell subsets in transcription factors and cytokine profiles, bridging innate and adaptive immunity. Patients with RA exhibit increased ILC1 in synovial fluid, tissues, and lymph nodes [156,157]. ILC2-derived IL-4/IL-13 suppresses macrophage production of IL-1β and TNF-α; adoptive transfer of ILC2 ameliorates arthritis in mice, indicating regulatory potential [158]. ILC3—comprising NKp46^+^ and CCR6^+^ lymphoid tissue inducer (LTi)-like subsets—is a major source of IL-17 and IL-22 in mucosal sites. Synovial ILC frequency correlates with joint tenderness and swelling [159]. ILC3 expands in early RA but not in healthy or high-risk individuals. In CIA mice, CCR6^+^ ILC3 infiltrates joints with elevated IL-17A and IL-22 [159]. In SKG mice, IL-2, IL-33, and CpG DNA from inflammatory cells activate synovial ILCs to produce granulocyte-macrophage colony-stimulating factor, initiating spontaneous arthritis [160], supporting a gut–joint axis.
2. Mucosa-associated invariant T (MAIT) cells: These RORγt^+^CD3^+^CD4^−^CD8^−^ T cells reside predominantly at mucosal surfaces and respond to microbial metabolites, linking gut microbiota to immunity [161,162]. Patients with RA show reduced peripheral blood MAIT cells but increased accumulation in synovial fluid, implicating their pathogenic role [163]. In CIA and collagen antibody–induced arthritis models, MAIT cell transfer into major histocompatibility complex class I-related molecule 1 knockout (MR1^−/−^) mice worsens disease [164], while MAIT-cell-deficient mice exhibit milder CIA—reversed upon MAIT cell reconstitution [165]. Although their joint homing origin remains unclear, their mucosal enrichment suggests possible migration from the gut during inflammation [166].
3. Intestinal Tfh cells: Defined by high CXCR5 expression, Tfh cells migrate toward CXCL13 to germinal centers, where they secrete IL-21 to drive B cell differentiation and IgG production [167,168]. Aberrant Tfh cell activity contributes to RA: Patients show elevated Tfh cells in blood and synovium [169], and IL-21 levels correlate with DAS28 scores [170,171]. In K/BxN B cell lymphoma 6 (Bcl-6)–deficient mice, antibiotic treatment reduces Tfh cells in the spleen, inguinal, and mesenteric lymph nodes, demonstrating microbiota-dependent Tfh cell regulation of arthritis [172]. SFB promote systemic Tfh cell differentiation via Bcl-6, exacerbating disease. Conversely, follicular regulatory T (Tfr) cells—CD4^+^CXCR5^+^Foxp3^+^—suppress Tfh and B cell responses in germinal centers [173]. A decreased Tfr/Tfh cell ratio in blood is linked to RA immunopathogenesis [174], and SFB can disrupt this balance, further fueling autoimmunity [175].

In RA, metagenomic and metabolomic studies show that an imbalance between butyrate-producing and butyrate-consuming gut bacteria correlates with anti-cyclic citrullinated peptide (anti-CCP) antibody levels and joint deformities [176]. *Fusobacterium nucleatum*, enriched in patients with RA, secretes outer membrane vesicles containing the virulence factor fibronectin-binding adhesin A (FadA), which are delivered to joints and activate synovial macrophages via the Ras-related protein Rab-5a (Rab5a)–Y-box binding protein 1 (YB-1) axis, driving inflammation [177]. Targeting microbial metabolites in FLS ameliorates RA through the HDAC3–forkhead box K1 (FOXK1)–IFN pathway [178]. Gut-microbiota-derived indole also promotes CIA in mice [179]. Gut dysbiosis and associated serum metabolome alterations are already evident in high-risk individuals with RA-associated autoantibodies, disrupting key metabolic pathways [180]. Transplantation of such dysbiotic microbiota impairs intestinal barrier integrity and triggers Th17-cell-mediated mucosal immune dysregulation, suggesting that early gut ecosystem disruption may initiate RA during the preclinical phase. This highlights the potential of microbiota monitoring or modulation for RA prevention in at-risk populations. Supporting this, a University of Leeds study [181] found that individuals with genetic, environmental, or immunological risk factors exhibit significant gut microbiome shifts—particularly in *Prevotellaceae*—prior to clinical onset. Moreover, *C. aerofaciens* enhances preclinical autoantibody production and disease severity, likely by increasing gut permeability and promoting systemic inflammation [182]. In conclusion, future gut microbiota interventions targeting zonulin, SCFAs, or lymphocyte migration (such as sphingosine 1-phosphate inhibitors) in patients with RA show great promise, but mechanistic precision is required. The field must address cohort heterogeneity (differences in disease stages, treatments, and microbiota composition) to establish clear causality (Box 2).

Box 2. Gut microbiota and microbial metabolites in RA pathogenesis

SCFAs

• Protective effects: Enhance gut barrier integrity by up-regulating TJs
• Therapeutic potential: Dietary fiber increases SCFA levels, reducing RA inflammation
• Clinical correlation: Depletion of butyrate-producing bacteria linked to anti-CCP antibodies and joint damage

Tryptophan metabolites

• Dual roles: Certain indoles improve gut barrier function, while others exacerbate CIA
• Mechanistic insight: Modulating HDAC3–FOXK1–IFN axis improves RA symptoms
• Clinical marker: Reduced circulating kynurenine levels observed in patients with RA

Bile acids

• Barrier regulation: Exhibit variable effects on intestinal permeability
• Synovial deficiency: Decreased levels in RA synovial fluid
• Therapeutic approach: Dietary reduction of serum bile acids alleviates RA-associated pain
• Pathogenic mechanism: *F. nucleatum* secretes FadA-containing vesicles, activating synovial macrophages via Rab5a–YB-1

Recent research identifies pronounced rhythmicity in gut microbiota, which modulates host genetics, metabolism, and immunity. Unhealthy lifestyles (irregular eating/sleep) disrupt this rhythmicity to drive disease. A study [183] showed dietary rhythms regulate gut microbiota diurnal oscillations, thereby shaping RA immune rhythms—enriching RA temporal medicine theory and providing new diagnostic/therapeutic strategies. In RA gut microbiota systems biology, a study [184] established the first RA gut microbiota resource library, containing 601 strains from 280 species (43 newly described). It also identified 20 RA-associated core bacterial species that influence host inflammation and autoimmunity; the most relevant species (*Mediterraneibacter tenuis* and *Agathobacter rectalis*) exacerbated murine inflammatory responses, offering valuable resources for RA etiology research and new therapy development. For RA treatments, sinomenine alleviates inflammation and immune responses by enhancing indole-tryptophan metabolites from specific gut bacteria, activating the AhR, and regulating NF-κB/mitogen-activated protein kinase pathways [185]. Photobiomodulation therapy (PBMT) exerts therapeutic effects on RA via the gut–joint axis, with specific amino acid metabolites (proline and *N*-acetyl-aspartate) playing key roles dependent on target organ enzymatic activity [186]. Another study first reported the efficacy and mechanisms of *Parabacteroides distasonis* (as probiotics) and ginsenoside Rg2 (as prebiotics) in RA treatment [187]. In addition, a high-fiber diet synergizes with *P. copri* to aggravate RA [188], highlighting the importance of ecological balance in evaluating dietary intervention impacts on RA pathogenesis and providing new insights for RA management via diet or microbiome modulation.

**Fig. 3.**
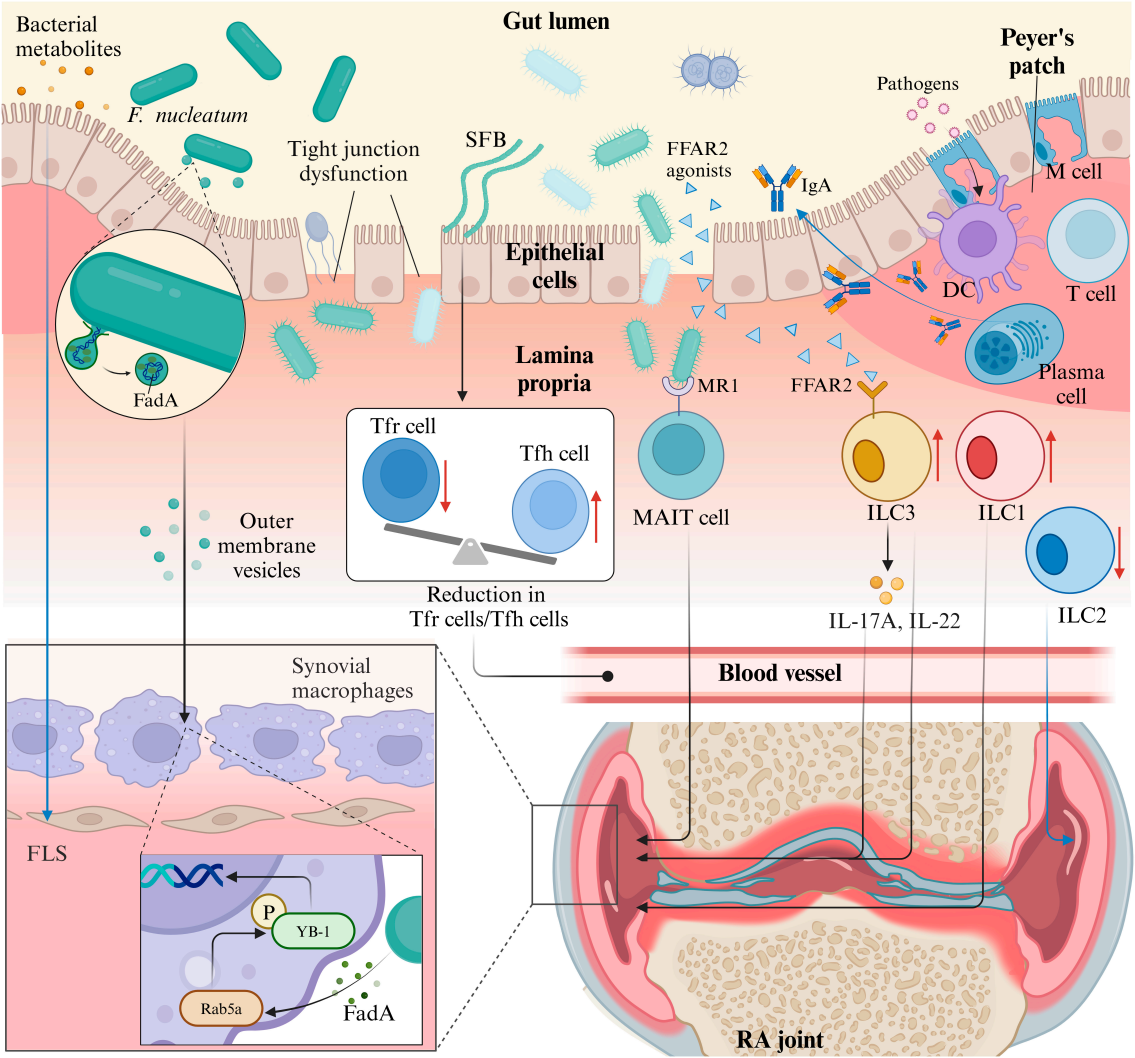
Gut microbiota in RA. In RA, intestinal mucosal immune regulation is critical. IgA, produced in Peyer’s patches, prevents pathogen penetration through the intestinal barrier. Barrier compromise allows DCs to recognize bacteria as antigens, triggering inflammation. Immune cells such as ILC1 and ILC3 increase in RA, while ILC2 decreases. ILC3 cells sense FFAR2 agonists, producing IL-17A and IL-22 that affect joints. MAIT cells detect bacterial components via MR1 receptors, influencing arthritis development. SFB disrupt the Tfr/Tfh cell balance, reducing their ratio. In addition, *F. nucleatum* activates synovial macrophages through outer membrane vesicles containing FadA, promoting joint inflammation via the Rab5a–YB-1 axis. Blue lines denote beneficial effects, and black lines indicate pathogenic mechanisms. This figure was created using BioRender.com. MR, mannose receptor.

In summary, the intestinal mucosal immune system resists gut microbiota invasion. Disrupted intestinal homeostasis (commensal-pathogen imbalance, mucosal barrier damage, and inflammation) enables bacterial lamina propria invasion, triggering abnormal immune activation that spreads to other organs and causes immune-related diseases. While “gut–brain” and “gut–lung” axes underscore gut microbiota’s role in extraintestinal diseases, RA-specific evidence indicates that gut dysbiosis precedes disease onset and influences progression, emphasizing the gut–joint axis. Understanding these mechanisms may reveal new therapeutic targets: Restoring microbiota balance via probiotics/prebiotics promotes anti-inflammatory functions (e.g., SCFA and tryptophan metabolite production). Pathogenic microbiota disrupts the mucosal barrier via CXCR3 and myeloid differentiation primary response 88 (MYD88) signaling, inducing local immune activation and joint inflammation, highlighting the need to correct this process (Fig. 3).

### Type 1 diabetes mellitus and gut microbiota

Type 1 diabetes mellitus (T1DM) is an autoimmune disorder driven by pancreatic β cell destruction, resulting in insulin deficiency. It accounts for 5% to 10% of diabetes cases, predominantly in children and young adults [189]. In 2019, over 1.1 million individuals under 20 lived with T1DM globally, with approximately 128,900 new annual cases in this age group [190]. Although genetic susceptibility is a key factor, fewer than 10% of at-risk children develop T1DM, underscoring the critical role of environmental triggers—such as diet, viral infections, and antibiotic exposure—in initiating autoimmunity [191,192]. T1DM evolves through 3 stages: initiation (autoantibody positivity with normoglycemia), acceleration (progressive β cell loss and dysglycemia), and clinical onset (severe insulin deficiency requiring therapy) [193]. Early interventions—including genetic screening, probiotics, and immune modulation—hold preventive potential [194].

In patients with T1DM, the gut microbiota acts as a pivotal environmental modulator of T1DM pathogenesis. Autoantibody-positive individuals show increased *Bacteroidetes* and reduced *Firmicutes* relative to healthy controls [195], alongside diminished SCFA-producing taxa—a pattern consistently reported in recent studies [196]. In genetically susceptible children, reduced microbial diversity and elevated *Bacteroidetes* correlate with pancreatic autoimmunity [197,198]. A landmark Swedish infant cohort linked fecal microbiota composition at 1 year of age (the typical window for islet autoimmunity onset) to HLA genotypes, revealing the first human evidence of HLA–microbiota interactions in T1DM [199]. Integrating machine learning with multiomics, Tan et al. [200] further demonstrated coordinated dysregulation across the microbiome, metabolome, and lipidome in patients with T1D. Functional evidence from animal studies exploring the gut microbiota’s involvement in T1DM pathogenesis supports a compelling mechanistic link: FMT from nonobese diabetic (NOD) mice induces insulitis in otherwise resistant recipients [201,202]. Collectively, these findings position the gut microbiota as a promising target for T1DM prevention. Given the developmental and anatomical proximity between gut and pancreas, it is hypothesized that gut-derived microbes or immune cells may access pancreatic tissue, fostering β cell autoimmunity [203–207].

#### Gut microbiota and intestinal mucosal barrier dysfunction in T1D

The integrity of the intestinal mucosal barrier—comprising mechanical and immune components—is critical for maintaining systemic immune homeostasis. The role of gut microbiota in promoting or inhibiting T1DM development is tightly linked to its ability to disrupt or restore intestinal mucosal barrier function [208].

**Fig. 4.**
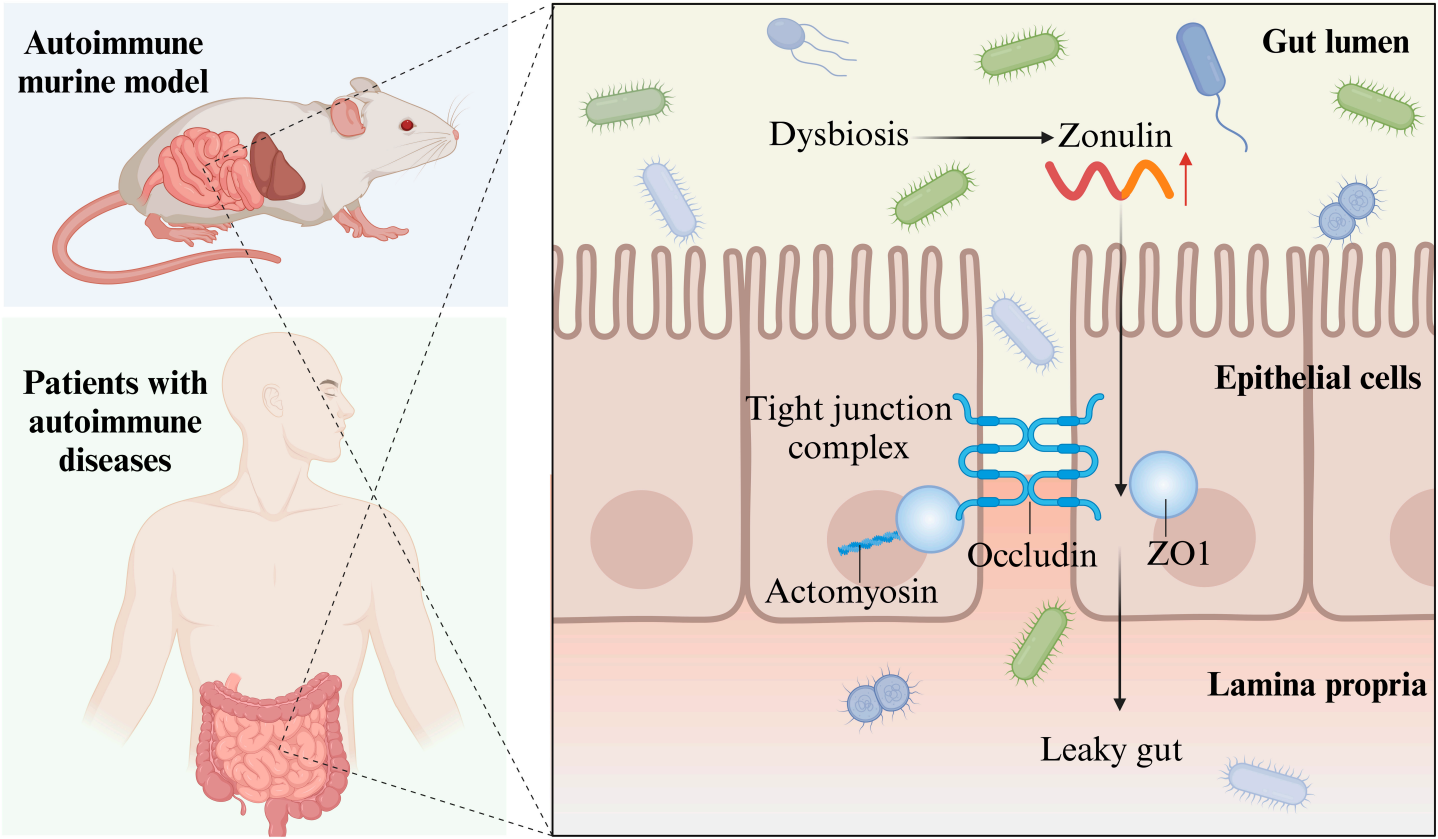
Alteration of gut permeability in AIDs. Changes in gut permeability have been found in various AIDs, including T1DM, RA, and multiple sclerosis. Evidence from both autoimmune murine models and patients with AIDs indicates that, during dysbiosis, the integrity of the gut barrier can be compromised because of the activation of zonulin, which disrupts TJ proteins. Zonulin features an epidermal-growth-factor-like motif and a peptide that activates proteinase-activated receptor 2. Zonulin activation disrupts TJs, causing the displacement of ZO1 and occludin from the junctional complex. This reduces gut barrier permeability, a condition often referred to as “leaky gut”. This figure was created using BioRender.com.

1. Pathogenic gut bacteria drive T1DM via zonulin: The integrity of the intestinal mucosal mechanical barrier is associated with zonulin production in small intestinal epithelium [209]. Zonulin regulates intestinal mucosal permeability, contributing to T1DM onset [209,210]. Gut dysbiosis strongly stimulates zonulin release: Toxins from certain pathogenic or conditionally pathogenic bacteria (e.g., *Escherichia* and *Enterococcus*) up-regulate zonulin via the MyD88-dependent pathway, damaging intestinal mucosal TJs and increasing intestinal permeability [209]. This allows more pathogenic bacteria and endotoxins to enter the body, initiating local intestinal inflammation and contributing to AID such as T1DM—a pathological process termed “leaky gut syndrome” [208]. Zonulin blockers (e.g., larazotide acetate) reduce intestinal permeability and T1DM risk in BioBreeding rats by down-regulating zonulin [211]. Plasma zonulin levels are higher in high-risk individuals with autoantibodies against ≥2 islet antigens than in patients with T1DM with negative or single-positive autoantibodies [211], indicating that elevated plasma zonulin is an early biomarker for screening T1DM high-risk individuals and a potential target to inhibit/delay T1DM onset and progression (Fig. 4).
2. IgA-coated gut microbiota trigger islet immune responses: Secretory IgA (sIgA) is an intestinal mucosal immune barrier effector molecule, synthesized and released by Peyer’s patch B cells [212]. Patients with newly diagnosed T1DM have higher proportions of sIgA-coated gut microbiota (*Escherichia*, *Enterococcus*, and strict anaerobes) than healthy individuals [212]. Fecal transplantation from T1DM donors to GF mice increases sIgA-bound gut microbiota in plasma/feces, reduces colonic/fecal free sIgA, and alters immune effector cells: decreased IgA + B cells in pancreatic draining lymph nodes, elevated CD4T/CD8T ratio/activity, and reduced intestinal lamina propria Treg cells [212]. After sIgA binds to gut microbiota, it drives epitope expansion, activates innate immunity in GALT, and initiates or exacerbates islet autoimmunity. In children with susceptible HLA genotypes (DR3–DQ2 and DR4–DQ8), sIgA-coated gut microbiota correlates with islet autoantibody types and titers: e.g., *Roseburia faecis*, *Streptococcus gallolyticus*, *Enterococcus faecalis*, and *Lacticaseibacillus acidophilus* associate with insulinoma-associated antigen-2 autoantibodies (IA2A), while sIgA-coated *B. fragilis* and *Ruminococcus* associate with glutamic acid decarboxylase autoantibodies (GADA) [213]. Higher proportions of sIgA-coated gut microbiota in patients with T1DM correlate with higher islet autoantibody titers and greater pancreatic β cell destruction [214]. Thus, in HLA-susceptible individuals, specific sIgA-coated gut microbiota (*Roseburia*, *Lactobacillus*, *B. fragilis*, and *Ruminococcus*) serve as an additional early marker for islet autoimmunity initiation/progression, alongside islet autoantibodies. Diet modulates sIgA-coated gut microbiota proportions in patients with T1DM or GF mice. Supplementation with SCFAs reduces pathogenic sIgA-coated bacteria (*E. coli* and *B. fragilis*) and increases sIgA-coated beneficial probiotics (*Lactobacillus* and *Akkermansia*) [215]. In GF mice receiving pathogenic fecal transplants, supplementation of SCFAs decreases sIgA-coated gut bacteria, reduces CD4^+^ T cells, increases Treg cells, and alleviates islet inflammation [212], suggesting that SCFAs mitigate islet autoimmunity by modulating sIgA-coated gut microbiota proportions.

#### Key effect pathways of gut microbiota regulation on T1DM

In the context of gut microbiota dysregulation and the NOD-like receptor pyrin domain-containing protein 3 (NLRP3) inflammasome: As a key member of the NOD-like receptor family, NLRP3 mediates antigen recognition. The NLRP3 inflammasome acts as a critical sensor for intestinal inflammatory signals and drives pancreatic autoimmune responses [216]. LPS—a major bacterial virulence factor—primarily activates NLRP3, while SCFAs, metabolites of probiotics such as bifidobacteria and *Akkermansia*, suppress NLRP3 activation [216,217]. During gut dysbiosis, pathogen-associated molecular patterns activate innate immunity via TLRs, creating an intestinal inflammatory microenvironment. This triggers the LPS–TLR4–IL-1 receptor-associated kinase–TNF receptor-associated factor 6–TGF-β-activated kinase 1–NF-κB signaling cascade, ultimately up-regulating never in mitosis gene A-related kinase 7 (NEK7) [218]. NEK7 induces cellular potassium efflux, elevating NLRP3 expression and activation; this, in turn, activates caspase-1, enhancing synthesis and secretion of IL-1β and IL-18 [24]. IL-1β is closely linked to early T1DM progression: It mediates pancreatic autoimmunity, recruits additional proinflammatory factors to islets, and exacerbates islet cell apoptosis [219].

1. Gut microbiota dysregulation and NETs: NETs form following neutrophil apoptosis. When intestinal mucosal barrier function is impaired, certain pathogenic gut bacteria (e.g., *Proteobacteria* and *Bacteroidetes*) recruit neutrophils to the pancreas via circulation. These neutrophils generate reactive oxygen species and activate peptidyl-arginine deiminase 4 (PAD4), leading to excessive NET formation [220]. NETs stimulate antineutrophil cytoplasmic antibody (ANCA) production; ANCAs cross-react with islet antigens to trigger or worsen islet immune damage [220]. In patients with T1DM, circulating neutrophils are reduced, while pancreatic neutrophils and NETs increase locally. PAD4 deletion in *Proteobacteria*-dominant NOD mice reduces NETs and alleviates/reverses islet inflammation [221,222], indicating that the PAD4–NET pathway is a key mechanism by which gut microbiota enhances intestinal innate immunity and subsequent pancreatic autoimmunity.
2. Gut microbiota dysregulation and ILC3: ILC3s, enriched in GALTs, are key mediators of defense against pathogenic microbes [223]. Mature ILC3 express multiple receptors, with free fatty acid receptors (FFARs) and AhRs being critical [223]. SCFAs—metabolic by-products of gut probiotics—act on FFAR or AhR to activate ILC3, which inhibits T1DM onset via 2 mechanisms: (a) maintaining intestinal mucosal barrier integrity by secreting IL-22 (promoting epithelial repair and preserving TJs) and (b) suppressing immune responses via IL-2 (enhancing intestinal Treg cell proliferation and immunosuppressive function, ultimately reducing islet β cell immune destruction) [223]. Transient intestinal infection with pathogenic bacteria (e.g., *Citrobacter rodentium*) enhances ILC3 proliferation, supporting long-term mucosal defense [224]. Thus, gut dysbiosis activates ILC3’s immunoregulatory function, which may act as a negative regulatory mechanism to counteract enhanced intestinal innate immunity and subsequent pancreatic autoimmunity.
3. Bidirectional regulation of pancreatic immune responses by gut microbiota at the molecular signaling pathway level: (a) pathogenic-gut-bacteria-induced signaling pathways for pancreatic autoimmunity: TLRs play a pivotal role in pathogenic bacteria-mediated pancreatic autoimmune damage, among which TLR4–LPS binding is critical. Gut pathogens (via LPS) activate the TLR4/MyD88/NF-κB pathway, up-regulating NLRP3 and recruiting monocytes, T cells, and natural killer (NK) cells to infiltrate islets, exacerbating pancreatic β cell autoimmune damage [225]. MyD88 blockade in NOD mice reduces the risk of autoimmune pancreatic inflammation [226], indicating that the TLR4/MyD88/NF-κB pathway is critical for transmitting enhanced intestinal innate immunity to pancreatic autoimmune escalation. (b) Probiotic-mediated signaling pathways inhibiting pancreatic autoimmunity: Probiotics inhibit pathogenic-bacteria-induced intestinal innate immunity and pancreatic autoimmune escalation mainly via the FFAR signaling pathway in ILC3. FFAR2 (a G-protein-coupled receptor) binds SCFAs (acetate and propionate), couples to G_i_/o or G_q_ proteins, and induces phosphorylation of mitogen-activated protein kinase and activating transcription factor 3—this elevates IL-22 expression in colonic ILC3, which, in turn, suppresses the TLR4/MyD88/NF-κB pathway. In addition, it up-regulates forkhead box protein P3 (Foxp3) expression, promoting intestinal Treg cell proliferation and increasing IL-10 levels, which also inhibits pancreatic autoimmunity [225,227].

**Fig. 5.**
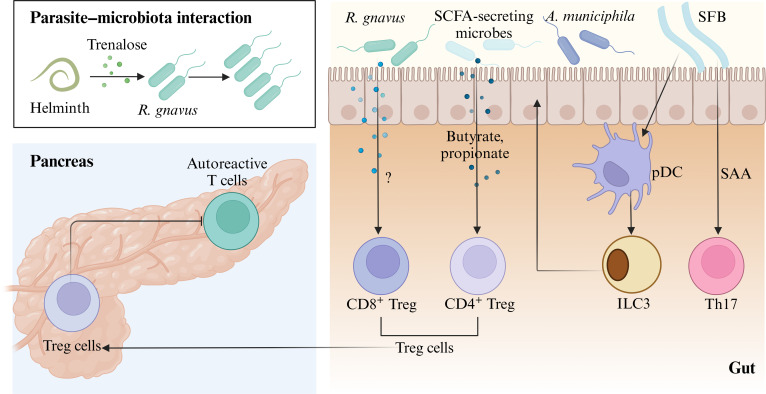
The role of the gut microbiota in the pathogenesis of T1DM. The parasitic worm *Heligmosomoides polygyrus* inhibits T1DM in mice by secreting trehalose, which increases *Ruminococcus* abundance. This bacterium induces Treg cell differentiation in pancreatic lymph nodes, suppressing autoreactive T cells and slowing T1DM progression. Patients with T1DM often have fewer CD8^+^ Treg cells and reduced *Ruminococcus* levels. Gut microbes produce SCFAs that promote CD4^+^ Treg cell differentiation, further suppressing autoimmunity. Reduced *Akkermansia* increases intestinal permeability, worsening disease. In animal models, SFB colonization activates Th17 cells and IL-22-producing ILC3s, with IL-22 strengthening the epithelial barrier. This figure was created using Biorender.com. SAA, serum amyloid A.

In conclusion, specific sIgA-coated gut microbiota can act as a biomarker supplementary to pancreatic islet autoantibodies; their combined detection enables accurate prediction of T1DM progression. Elevated serum zonulin is an early biomarker for screening T1DM high-risk individuals and a potential target to inhibit/delay T1DM onset and progression. Dietary fiber, oral probiotics, or their metabolites (e.g., SCFAs) can improve gut microbiota composition, regulate key pathways, inhibit islet autoimmunity, and lower T1DM risk. Future large-sample, multiethnic clinical intervention studies are required to validate the mechanisms linking gut microbiota dysbiosis to pancreatic autoimmune damage (Fig. 5 and Box 3).

Box 3. Gut microbiota and microbial metabolites in T1DM pathogenesis

Dysbiosis features

• Patients with T1D exhibit reduced β-diversity, particularly in those with albuminuria
• The abundance of SCFA-producing bacteria such as *A. rectalis* is significantly decreased
• Pathogenic bacteria including LPS-producing species and *Clostridium bolteae* show increased proliferation

Metabolite alterations

• Tryptophan metabolism is disrupted, with elevated plasma indoxyl sulfate and l-kynurenine alongside reduced tryptophan levels in patients with macroalbuminuria
• Adult patients with T1D demonstrate decreased phenolic acid derivatives
• Altered lipid metabolism features increased glycerophospholipids and branched-chain amino acid (BCAA) derivatives, which correlate with diabetes risk
• FMT intervention leads to elevated eicosapentaenoic acid and docosahexaenoic acid levels that improve the testicular microenvironment

Microbiome-disease links

• Reduced *Lachnospiraceae* (S24-7) abundance impairs Reg3γ expression and accelerates T1D progression
• FMT treatment decreases CXCR3^+^ T cell populations, thereby preserving residual β cell function
• Disrupted bacteriophage–bacteria interactions contribute to intestinal barrier dysfunction

Intervention strategies

• Autologous FMT demonstrates superior efficacy in preserving stimulated C-peptide levels compared to heterologous FMT
• Maternal microbiota transplantation prevents antibiotic-accelerated T1D development in NOD mice
• Alginate oligosaccharide supplementation enriches beneficial *Bifidobacterium* populations and improves semen quality parameters
• Dietary modification through reduced sugar-sweetened beverage intake helps restore *Agathobacter eligens* populations and normalizes BCAA metabolism

#### Gut microbiota-based interventions for T1D improvement

Probiotics and FMT show promise for treating T1DM. One study showed improved glycemic control in children on probiotics, with reduced hemoglobin A1c (HbA1c) and insulin requirements [227]. Kumar et al. [228] conducted a randomized controlled trial, showing that FMT preserves β cell function in patients with newly diagnosed T1DM. Recent meta-analyses/systematic reviews indicate that probiotic supplementation improves HbA1c in T1DM, supporting gut-microbiota-based interventions as complementary therapies [204,229]. For FMT, a recent study [230] first established a well-characterized T1DM cohort to investigate gut microbiota and secondary bile acid dysfunction in T1D. Eligible participants received washed microbiota transplantation—functional microbiota from healthy donors to reconstruct patients’ gut microbiomes. It found that T1D correlates with reduced gut microbial diversity and a harmful/beneficial bacterial imbalance; patients with T1D also had impaired secondary bile acid metabolism. The study further confirmed that gut microbiota/metabolite-targeted interventions were safe, improved glycemic control, reduced daily insulin doses, and alleviated inflammation. They reshaped gut microbiota toward a healthier profile and promoted secondary bile acid production. Responders had increased beneficial bacteria/secondary bile acid levels and improved C-peptide responses. Overall, targeted modulation of gut microbiota and secondary bile acid metabolism may be a promising T1DM therapeutic approach.

### SS and gut microbiota

SS is a common multifactorial autoimmune and rheumatic disease (AID/RID) with definitive diagnosis remaining challenging [231]. Patients suffer from considerable clinical discomfort, highlighting the need to explore its pathogenesis, severity, early diagnosis, and treatment [232]. SS is characterized by lymphocytic infiltration of lacrimal/salivary glands (impairing glandular function) and diverse manifestations. Pathologically, salivary glands show infiltration of DCs, B/T lymphocytes, NK cells, and macrophages [233–235]. Autoantibodies in salivary glands trigger aberrant immune responses, with infiltrating inflammatory cells extensively damaging epithelial cells, leading to acinar atrophy, salivary duct loss, and progressive impairment of secretory function (causing xerostomia) [235–237]. Studies confirm close links between gut microbiota alterations and severe SS symptoms [238,239]. In patients with primary SS (pSS), proinflammatory gut microbiota levels increase while anti-inflammatory ones decrease. Patients with pSS also have distinct metabolic profiles, with specific associations between microbiota and metabolism [11]. Beyond disrupting gut microbiota abundance, pSS impairs host metabolic balance. Gut-microbiota-associated metabolites may shed light on pSS mechanisms and serve as potential tools for early prediction, diagnosis, and treatment [240].

#### Intestinal microbiota characteristics of pSS

In early life, gut microbiota drives the development of innate and adaptive immunity [241,242]; in adulthood, it continuously interacts with the immune system to maintain homeostasis. The innate immune system engages with microbiota via pattern recognition receptors (e.g., TLRs): Epithelial cells, ILCs, and DCs detect microbial antigens/metabolites to coordinate host–microbiota interface responses [141]. Activated symbiotic microbiota induce DCs to secrete IL-12, IL-15, and IFN, which act on NK cells to combat pathogens [243]. Gut dysbiosis—defined as an imbalance in microbial composition (often involving the *Firmicutes*/*Bacteroidetes* ratio)—is common in pSS pathogenesis, with significant changes in α- or β-diversity. α-Diversity reflects intrahost microbial variability (e.g., species richness/abundance differences), and β-diversity denotes intercommunity variations between diseased and healthy hosts [244]. Alterations in the host–microbial symbiotic network modify microbial virulence and metabolism; shifts in species relative abundances also constrain co-occurrence networks, inducing/excluding species or rearranging the network entirely [245].

Comparative analysis shows significant gut microbiota alterations in patients with pSS versus healthy individuals [246]. *Firmicutes* remain dominant (40% to 60%), followed by *Bacteroidetes*, *Actinobacteria*, and *Proteobacteria*; shifts in the *Firmicutes*/*Bacteroidetes* ratio are an early sign of gut ecological imbalance in pSS. At the phylum level [240,247], *Bacteroidetes*, *Proteobacteria*, and *Actinobacteria* increase, while *Firmicutes* decrease; orders *Clostridiales* and *Bacteroidales* decrease. At the family level, *Actinomycetaceae*, *Lactobacillaceae*, and *Coriobacteriaceae* increase, whereas *Lachnospiraceae* and *Ruminococcaceae* decrease. At the genus level, *Megasphaera*, *Parabacteroides*, and *Prevotella* increase, while *Faecalibacterium* and *Veillonella* decrease; the abundance of streptococci, *Bacteroides*, enterococci, and *E. coli* is lower than in healthy individuals. Notably, patients with pSS have increased proinflammatory microbes (e.g., *Shigella*, *Clostridium*, *Streptococcus*, and *Lactobacillus*) and reduced anti-inflammatory microbes (e.g., *Butyricimonas*, *Bulleidia*, and *Akkermansia*) [246]; elevated *Shigella*, *E. coli*, *Campylobacter*, and *Streptococcus* are confirmed pSS microbiota features. α-Diversity (intracommunity evenness/abundance) and β-diversity (intercommunity similarity at ecological distances) [248] show partial overlap between pSS and healthy gut microbiomes, but pSS is associated with fluctuating microbiota, elevated fecal-calcium-binding protein (indicating gut inflammation severity), and clinical symptom correlations: *Bacteroidetes* [247], *Proteobacteria*, and *Bifidobacterium* link to dry eye; *Actinobacteria* and *Prevotella* affect tear secretion, tear breakup time, and gut innate immunity (intestinal epithelial cells, ILCs, and DCs). A meta-analysis [240] revealed that patients with pSS have slightly reduced α-diversity versus healthy controls (lower Shannon–Wiener index, Chao1 richness, and abundance-based coverage estimator [ACE] index) and significant β-diversity differences, as well as *Firmicutes* depletion, *Proteobacteria* enrichment, and altered SCFA-producing microbes (*Ruminococcaceae*, *Lachnospiraceae*, *Faecalibacterium*, *Butyricicoccus*, and *Anaerobutyricum hallii*). Specific microbe/metabolite abundance and functional changes contribute to pSS pathogenesis, with some microbes correlating with clinical parameters.

In summary, pSS gut dysbiosis is characterized by reduced diversity, proinflammatory microbe enrichment, and anti-inflammatory microbe depletion; future research should clarify microbiota–pSS causal relationships. Intestinal dysbiosis triggers abnormal B cell diversification and Treg/Th17 cell imbalance, driving autoimmune ocular diseases. Gut-microbiota–epithelial cell–mucosal immune cell interactions differ in local/systemic immunity; growing evidence links microbiota to pSS, offering novel treatment perspectives. Note that gut microbiota research requires large, comprehensive samples.

#### Mechanisms of pSS pathogenesis related to gut microbiota

The gut is recognized as an “active organ” [240]. Gut microbiota may influence anxiety and depression symptoms in pSS [248] and may drive SS development by regulating host immunity and colonic neurotransmitter secretion—affecting the autonomic nervous system and hypothalamic–pituitary–adrenal axis. Interactions between gut microbiota, epithelial cells, and mucosal immune cells are critical for local and systemic immunity.

Key features of SS-associated microbiota include reduced gut/oral diversity, which correlates negatively with clinical symptoms. Genome-wide association study in SS identify gut bacterial components (e.g., LPS) that stimulate TLR2/TLR4 on peripheral blood mononuclear cells, inducing IL-17 production [246]. Th1 cells secrete proinflammatory cytokines (IFN-γ, IL-1β, IL-6, and TNF-α), while Th17 cells produce IL-17 (a core mediator of autoimmunity with high plasticity) and drive chronic inflammation plus autoreactive B cell responses. Autoreactive B cells—key in SS pathogenesis—function as antigen-presenting cells and secrete autoantibodies (anti-Sjögren’s syndrome antigen A [SSA/Ro] and antigen B [SSB/La] antibodies and muscarinic acetylcholine receptor 3), attracting inflammatory cells via receptor signaling or complement activation. Th1/Th17 cells induce inflammation [249], promoting proinflammatory cytokine/chemokine synthesis by immune and epithelial cells; Th17 cells further drive B cell differentiation into plasma cells and autoantibody production. Th1 cell polarization and IFN-γ secretion also link to SS pathogenesis: Early in SS, IFN-γ stimulates macrophages/DCs to target gut microbiota, up-regulating major histocompatibility complex (MHC) expression [250], and is critical for autoinflammation and autoimmune responses. IL-12 is a potent inducer of Th1 cell polarization, while IFN-γ contributes to SS manifestations in lacrimal glands and ocular surfaces. SS-related genetic pathways/molecules include interferon regulatory factor 5, IFN signaling, NF-κB (for B cell activation), lymphocyte signaling, and antigen presentation. IFN-γ—key for lacrimal gland destruction and secretory dysfunction—reduces tear component secretion (e.g., Rab3D), up-regulates MHC class II expression and corneal epithelial precursor envelope synthesis in patients with SS, and promotes goblet cell loss, epithelial apoptosis, conjunctival keratinization, and eventual squamous metaplasia.

As noted earlier, butyrate is a major gut bacterial metabolite. A study [251] found significantly reduced butyrate-producing beneficial bacteria in patients with SS. Butyrate—key for colonic epithelial cell energy supply and intestinal barrier enhancement—declines with such reductions. Proinflammatory pathogenic bacteria [31] can disrupt the intestinal barrier, exacerbating SS-related inflammation by increasing proinflammatory cytokines, reducing anti-inflammatory IL-10 secretion, and down-regulating peripheral FOXP3 mRNA. Butyrate-producing bacteria and *Clostridium* also maintain mucosal Treg/Th17 cell immune balance and contribute to autoimmunity induction; Treg cells’ anti-inflammatory effects are critical for preventing chronic inflammation, and their loss drives autoimmune progression. Interactions between gut microbiota, epithelial cells, and mucosal immune cells influence local and systemic immunity, with microscopic evidence supporting these links [252–254]. In SS, proinflammatory factors (IL-6, IL-12, IL-17, and TNF) correlate positively with *E. coli* abundance. For causal validation, Wang et al. [255] used 2-sample Mendelian randomization to confirm causal associations between bacterial genera and SS: the *Coprobacillus* (formerly “*Eubacterium*”) *coprostanoligenes* group exerts protective effects by down-regulating CXCL6 in SS. Limitations include insufficient granularity for subgroup analyses in MiBioGen/FinnGen datasets (due to incomplete demographic information); multiomics validation is needed to clarify SS pathogenesis within long-term gene–environment interactions. Microbiota alterations drive pathology via 3 key pathways: molecular mimicry, metabolite alterations, and disrupted epithelial tolerance—ultimately causing immune dysregulation and clinical manifestations (dry eyes and dry mouth). Growing research on gut microbiota–SS links underscores microbiota’s critical role in SS, offering new perspectives for treatment (Box 4).

Box 4. Gut microbiota and microbial metabolites in pSS pathogenesis

Microbiome dysbiosis features

• Diversity reduction: Patients with pSS exhibit significantly decreased α-diversity in gut and oral microbiota compared to healthy controls
• Signature taxa: Patients with pSS show distinct gut microbiota changes, featuring highly abundant *Ligilactobacillus salivarius* (linked to inflammation), reduced *Ruminococcus torques* (unlike SLE), and shifted *Firmicutes*/*Bacteroidetes* ratios favoring *Bacteroides* species
• Functional alterations: pSS exhibits disrupted microbial pathways, particularly up-regulated l-phenylalanine biosynthesis and increased bacterial-invasion-related virulence genes

Multiniche microbial changes

• Oral microbiome: The oral microbiome shows the most pronounced alterations in pSS, with salivary flow reduction explaining 9% of microbial variation (versus 5% from disease status). These distinct changes, primarily driven by xerostomia, differ significantly from SLE patterns
• Gut–vagina Axis: Patients with pSS show coordinated microbial diversity loss across gut, oral, and vaginal sites, with dysbiosis often preceding clinical symptoms, suggesting pathogenic involvement rather than secondary effects

Immunomicrobial interactions

• Molecular mimicry: Gut microbiota harbors peptides mimicking pSS autoantigen epitopes
• Th17/Treg cell imbalance: pSS features reduced butyrate producers (impairing Treg cells) and elevated *E. coli* (linked to IL-6/IL-17/TNF-α increases)
• Barrier disruption: The activation of TLR2/4 by LPS stimulates IL-17 production, while pathogen overgrowth disrupts intestinal barrier function

#### Gut microbiota treatment strategies

pSS treatment studies focus on dietary intake, probiotics, and FMT, yielding diverse therapeutic strategies. Bacterial therapy—an emerging approach for autoimmunity [6]—acts by normalizing gut microbial communities to prevent/treat dysbiosis-induced AIDs. Strategies such as “nourishing Yin and benefiting Qi” to enhance gut microbial diversity/richness have also been explored [256]. In addition, metformin has been studied for pSS by enhancing immune regulation—via activating adenosine 5′-monophosphate-activated protein kinase [257]. White peony glucosides [258] increase fecal moisture (alleviating constipation), modulate gut microbiota, raise acetate/butyrate levels, up-regulate TJ proteins, and restore lesion immune function. While gut microbiota modulation studies have identified potential pSS treatment targets, few strategies are clinically feasible, posing major challenges for pSS treatment. In conclusion, unresolved issues remain in pSS research: Uncertainties persist regarding SS’s specific pathogenesis, and no studies on early-stage SS gut microbiota exist. Future research should clarify pSS etiology/pathogenesis and gut microbiota dynamics during disease onset; animal models of early disease (compared to healthy controls) are recommended to inform early detection/treatment and potentially guide other AID therapies. In summary, gut microbiota dysbiosis is common in pSS and linked to disease activity, characterized by reduced commensals and increased pathogens. This imbalance triggers B cell diversification (especially IgA production), intestinal mucosal damage, and antigen exposure, followed by excessive T cell activation and proinflammatory DCs. This initiates local mucosal immunity, further disrupts gut barrier function, and drives systemic inflammation/autoantibody production (contributing to arthritis and salivary gland infiltration). Modulating gut microbiota (via probiotics, prebiotics, antibiotics, or diets) may benefit pSS during onset/activity; understanding microbiota’s role in pSS pathogenesis could advance disease insights, therapies, and drug development.

## Interactions between Gut Microbiota and Therapeutic Drugs for AIDs

Tumor immunotherapies (e.g., programmed cell death protein 1 [PD-1] inhibitors) are linked to gut microbiota composition; modifying gut microbiota to reverse drug resistance can enhance therapeutic outcomes [259]. Gut microbiota and their enzymatic functions influence intestinal absorption/metabolism of immunomodulatory drugs (slow-acting antirheumatic drugs and biologics), potentially causing drug inactivation, abnormal activation, or toxic metabolite production—affecting efficacy, toxicity, and interpatient treatment variability. Studying the drug–microbiome axis may predict immune-modulating therapy efficacy; adjusting gut microbiota can optimize drug metabolism to enhance bioactivity or reduce toxicity. Methotrexate (MTX)—a cornerstone for RA (and used in SLE)—is metabolized by gut commensals with glutamate carboxypeptidase activity to inactive diamino-*N*^10^-methylpteroic acid. Artacho et al. [260] identified strong associations between gut microbiota (classification and abundance) and MTX clinical responses, indicating that microbiota influences MTX metabolism/effectiveness. Conversely, MTX inhibits host dihydrofolate reductase (DFR)—a conserved bacterial enzyme—causing off-target effects on bacterial DFR that disrupt intestinal bacterial proliferation, metabolism, and diversity [261]. Sulfasalazine (SSZ) has closer microbiota ties: It requires gut commensal azoreductase to release active 5-aminosalicylic acid and is inactivated by bacterial arylamine-*N*-acetyltransferase; its sulfonamide component has antibacterial effects altering microbiota composition. Commensals producing these enzymes (*Bacteroides*, *Lactobacillus*, *Enterococcus*, *Clostridium*, *Akkermansia*, and *Parabacteroides*) influence SSZ efficacy [262]. Cyclophosphamide immunogenicity is influenced by *Enterococcus hirae* and *Barnesiella intestinihominis*; hydroxychloroquine increases gut microbiota diversity/abundance (especially *Prevotellaceae*) [125,263]. Mycophenolate mofetil efficacy correlates with expanded *E. coli*/*Shigella* and reduced *Clostridium*, *Akkermansia*, and *Parabacteroides* [264], while commensals expressing β-glucuronidase increase its gastrointestinal toxicity [265]. Recent animal studies show that *L. acidophilus* supplementation modulates Treg/Th17 cell balance, reduces anti-dsDNA titers in lupus mice, improves renal pathology, and enhances tacrolimus efficacy, demonstrating microbiota interventions’ potential to boost traditional therapy outcomes [266]. In patients with PBC, poor UDCA response correlates with reduced *Faecalibacterium*, which may serve as a treatment response predictor [267].

Biologics have revolutionized AID treatment, with emerging evidence of gut–microbiota–biologic interactions. Elevated *Burkholderiales* levels partially predict TNF inhibitor (TNFi) efficacy in patients with SpA [268]. In AS patients, TNFi treatment partially restores gut *Bacteroides* abundance [269]. In IBD, TNF monoclonal antibody (e.g., adalimumab and infliximab) treatment reduces *Proteobacteria* and increases *Clostridiales*, correlating with favorable clinical outcomes [270]. Fecal metabolomics indicate that butyric acid and its synthetic substrate levels may inform TNFi efficacy evaluation in IBD [271]. Interactions between other biologics and gut microbiota remain under investigation. Future research aims to predict targeted biologic efficacy via individualized gut microbiome profiling and enhance biologic efficacy through microbiota interventions.

**Table 1.** Interactions between gut microbiota and AID therapies

Drugs	Diseases	Effects	Bacterial strains	References
MTX	RA, SLE	The abundance of the gut microbiota is related to the clinical effect of MTX	–	[260,261]
		Certain bacterial strains lead to off-target effects of MTX		
SSZ	IBD, AS	SSZ affects the composition of the gut microbiota	*Bacteroides*, *Lactobacillus*, *Enterococcus*, *Clostridium*, *Akkermansia*, and *Parabacteroides*	[262,263]
		Certain bacterial strains affects the therapeutic results of SSZ		
Cyclophosphamide (CTX)	SLE, PM, SCL	The immunogenicity of CTX can be affected by certain bacterial strains	*E. hirae* and *B. intestinihominis*	[262]
Hydroxychloroquine (HCQ)	RA, SLE, pSS	HCQ increases the diversity and abundance of the gut microbiota	*Prevotellaceae*	[262]
Mycophenolate mofetil (MMF)	RA, SLE	The therapeutic effects of MMF are related to the abundance of certain bacteria	*E. coli*/*Shigella*, *Clostridium*, *Akkermansia*, *Parabacteroides*, and β-glucuronidase-expressing bacteria	[264,265]
		Certain bacterial strains increase its gastrointestinal toxicity		
Tacrolimus (FK506)	RA, SLE	Certain bacteria strains enhance the efficacy of FK506	*L. acidophilus*	[266]
UDCA	PBC	Decreases in certain bacteria indicate response to UDCA	*Faecalibacterium*	[267]
TNFis	SpA, IBD	The abundance of certain bacteria indicates the efficacy of TNFi	*Burkholderiales*, *Proteobacteria*, *Clostridiales*, and butyric-acid-producing bacteria	[268–271]
		TNFi treatment affects the abundance of certain bacteria strains, which correlates with clinical outcomes		

## Interventions of Intestinal Microbiota in AIDs

Current gut microbiota interventions—including probiotics, prebiotics, FMT, limited known-strain bacterial transplantation, and dietary regulation—serve as adjunctive therapies for AIDs.

### Probiotics

*Lactobacillus* and *Bifidobacterium* are common, well-studied gut probiotics that support microbiota balance and reduce inflammation. For example, *Lacticaseibacillus casei* mitigates arthritis symptoms in CIA models by suppressing proinflammatory cytokine release [272]. In patients with RA, supplementation with *L. acidophilus*, *L. casei*, and *B. bifidum* improves DAS28 scores and reduces C-reactive protein (CRP) [273]. While *P. copri* links to RA pathogenesis [274], *Prevotella histicola* delays disease onset and reduces severity in HLA-DQ8-susceptible arthritis models [275]. A randomized double-blind trial shows that *Bacillus coagulans* alleviates RA symptoms, improves patient self-assessment, and lowers CRP [276]. However, gut microbiota complexity (microbial competition/symbiosis) and variations in disease type/stage, bacterial strains, and colonization sites mean no universal probiotic exists.

### Prebiotics

Prebiotics, which resist complete human digestion, act as nutrients for gut probiotics. Their fermentation promotes beneficial bacteria growth and SCFA production (e.g., oligosaccharides, fructooligosaccharides, and lactulose). They prevent pathogen epithelial adhesion/colonization, increase beneficial microbes (*Bifidobacterium* and *Lactobacillus*), suppress proinflammatory cytokines (IL-1, IL-6, and TNF-α), and promote anti-inflammatory IL-10, alleviating inflammation [277]. Examples are as follows: Oligosaccharides improve ulcerative colitis symptoms (urgency and diarrhea) [278]; inulin (rich in fructooligosaccharides) increases C-peptide levels of patients with T1DM and improves intestinal permeability [279]; composite prebiotics (lactulose + fructooligosaccharides) improve atopic dermatitis in high-risk infants [280]. While prebiotics benefit inflammation, effects are limited; probiotic–prebiotic combinations may enhance efficacy, but research in rheumatic diseases remains scarce.

### FMT and limited strain transplantation

FMT transplants fecal microbiota (including bacteriophages) from healthy donors to recipients to restore gut microbiota diversity/abundance, reshape microbial ecosystem stability, and improve intestinal permeability. Individual cases report improved disease activity in RA/psoriatic arthritis after FMT [281], but a double-blind randomized controlled trial found no superior FMT efficacy for psoriatic arthritis [282]. FMT research in autoimmunity faces challenges: Donor screening, fecal preparation, procedure standardization, and risk assessment need improvement. Limited strain transplantation—combining functionally known, limited bacterial strains—avoids FMT’s uncertainties/risks. Because of interindividual microbiota variability, optimal bacterial combinations differ by person and disease; more research is needed to refine combinations for personalized therapy.

### Dietary regulation

**Fig. 6.**
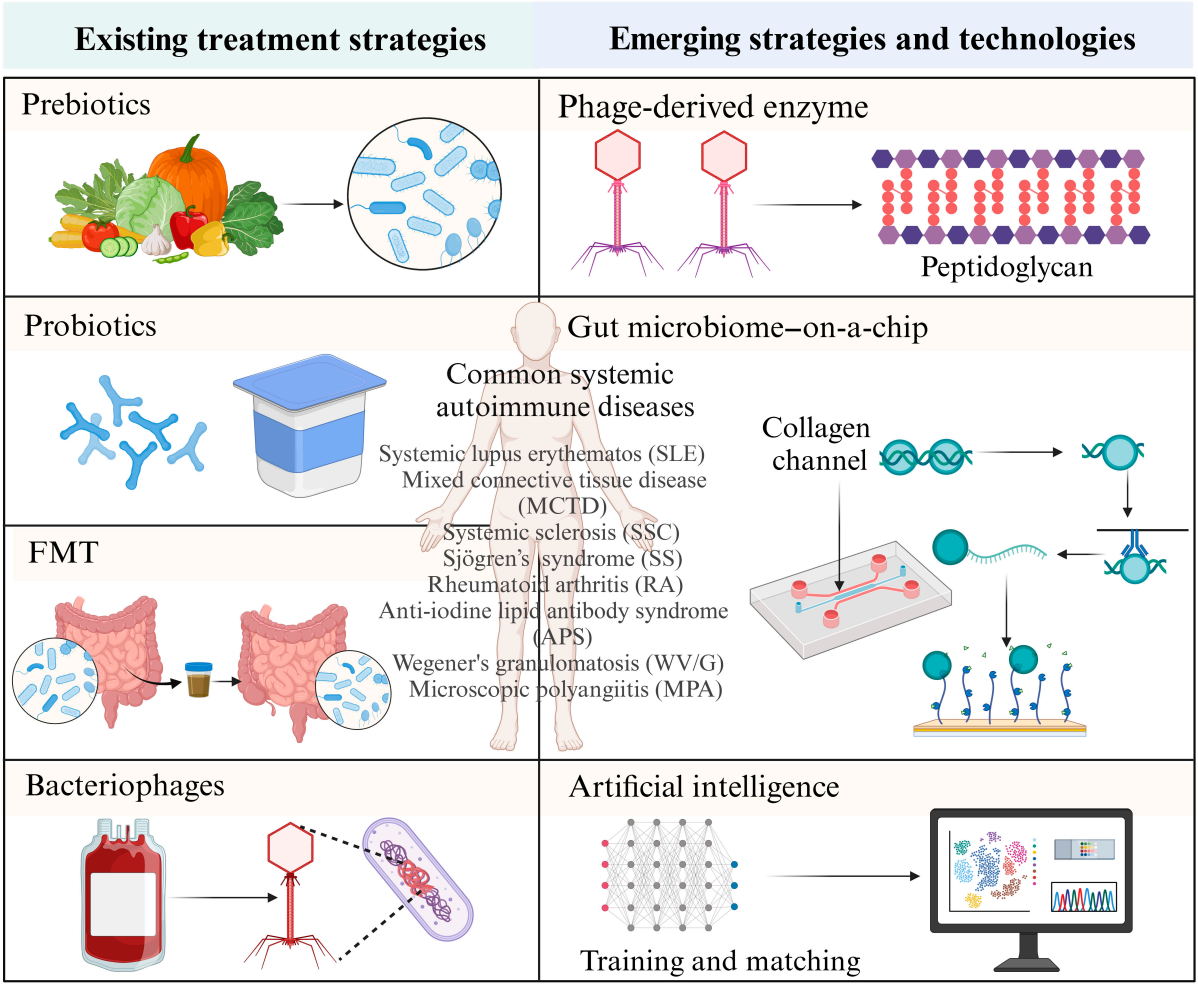
Existing treatment strategies and emerging strategies and technologies in the field of gut microbiota on AIDs. Strategies for modulating AIDs via gut microbiota include probiotics, prebiotics, FMT, and bacteriophages. Emerging technologies, such as phage-derived enzymes, gut microbiome–on-a-chip, and AI, are also advancing the field. Phage-derived enzymes, such as lysins, degrade bacterial cell walls selectively. Gut microbiome–on-a-chip mimics the intestinal environment for high-throughput screening, and AI analyzes microbial data to predict outcomes and optimize therapies. These approaches collectively enhance our understanding and treatment of AIDs. This figure was created using Biorender.com.

Diet provides carbon sources for gut microbiota, shaping their composition/metabolism. High-sugar/saturated fat diets cause dysbiosis and promote inflammation. The Mediterranean diet (high fiber/unsaturated fats) increases *Bacteroides*, reduces *Firmicutes*, lowers pathogenic *P. copri*, and boosts SCFAs (propionic acid and butyric acid) [283], exerting anti-inflammatory effects and improving RA activity. High salt intake reduces gut *Limosilactobacillus murinus*, promotes Th17 cell proliferation, and exacerbates Th17-cell-mediated autoinflammation [284,285]. Vitamin A intake provides retinoic acid, promotes *Lactobacillus* growth, and alleviates lupus-like phenotypes in lupus-prone mice [286]. Resistant starch-rich diets inhibit *Ruminococcus* growth/translocation and increase SCFAs, reducing autoimmunity [37]. With deeper understanding, dietary therapy may emerge as a key part of systemic treatment (Fig. 6).

## Summary

In recent years, the gut microbiota’s role in regulating immune homeostasis has been widely recognized. However, geographical variations, diverse sequencing methods, and differing treatments have resulted in inconsistent findings. Despite this, current research underscores the potential of gut microbiota studies to elucidate AID mechanisms, clarify variability in clinical and drug responses, and advance new therapies. Although research in this area is still in its early stages, future studies aim to combine detailed immune phenotyping with comprehensive genomic and functional analyses of gut microbiota across taxonomic levels (from phylum to strain). This integrated approach will help uncover the dynamic changes in gut microbiota during the onset and progression of AIDs. By investigating the links between gut microbiota, clinical phenotypes, and drug metabolism, researchers can use machine learning to create personalized microbiota profiles and predictive tools. These developments are expected to yield clinically relevant predictive models and tailored intervention strategies, ultimately enhancing treatment outcomes and patient prognosis.

In conclusion, the rapid development of high-throughput sequencing technologies has substantially advanced the study of gut microbiota genomics and metagenomics in AIDs. Research has revealed distinct differences in gut microbiota composition between patients with AIDs—such as RA [26,287], SLE [288,289], IBD [290], PBC [27], multiple sclerosis [291], AS [29,292], primary pSS [30], and Behcet’s disease [293]—and healthy individuals. These differences include reduced microbial diversity and the enrichment of disease-specific strains, whose abundance often correlates with disease activity. For instance, *P. copri*, *R. gnavus*, and *L. salivarius* are enriched in the fecal microbiota of patients with RA, with *L. salivarius* levels showing a positive correlation with disease activity [26,287]. Recent studies have shifted from merely describing structural differences in gut microbiota to investigating the pathogenic mechanisms of these microbial alterations. Notably, the role of microbial metabolites in regulating host immune homeostasis has garnered increasing attention.

## Future Challenges and Perspectives‌

### Challenges in fundamental research on gut microbiota

The first challenge lies in deepening and advancing the understanding of gut microbiota’s role in the mechanisms of AIDs. Future efforts must address the decoding of the complex interactions within the microbiota–host network. Although metagenomics has identified hundreds of microbiota features associated with AID/RID, such as the enrichment of *Prevotella* in patients with RA, most studies remain at the level of correlation. Beyond the known direct metabolite–receptor interactions, more complex indirect regulatory networks exist, including epigenetic regulation (e.g., microbial metabolites influencing histone modifications) and interorgan signaling (e.g., gut–liver and gut–brain axes). These multilayered interaction mechanisms are not yet fully understood, limiting the design of precise intervention strategies. Future research should focus on the following: (a) molecular-level interaction networks: investigating the direct mechanisms by which microbial metabolites (e.g., SCFAs and tryptophan metabolites) interact with host immune cells, particularly through signaling pathways such as AhR receptor and GPR43/41 and deciphering the molecular details of how microbial nucleic acids (e.g., bacterial DNA/RNA) activate autoimmune responses via pattern recognition receptors (TLRs and nucleotide-binding oligomerization domain-like receptors). (b) Immune-cell-subset-specific regulation: unraveling the dynamic modulation of Treg cells and Th17 cell differentiation by specific bacterial strains, as well as the trans-organ effects of gut-resident immune cells (e.g., γδ T cells) and expanding research on the “gut–organ axis” (e.g., gut–joint, gut–skin, and gut–brain axes) to explore communication mechanisms between gut microbiota and distant organs, particularly through novel extracellular vesicles, exosomes, and cytokine diffusion. The specific molecular mechanisms and dynamic changes of these cross-organ axes require further exploration [294–297]. (c) In terms of the complexity of epigenetic regulation, microbial metabolites not only directly activate receptors on immune cells but also regulate host gene expression through epigenetic mechanisms. For example, butyrate can enhance Treg cell function by inhibiting HDACs and promoting Foxp3 gene expression [298]. However, the tissue and cell type specificity of such regulation remains unclear. In addition, the roles of microbiota in DNA methylation and noncoding RNA regulation are emerging, but their specific contributions to AID/RID require further investigation.

Regarding the bottleneck in causality validation, most studies on microbiota–AID associations lack causal validation, with only a few addressing causality. Moreover, the reproducibility of these findings in human cohorts is questionable, particularly across diverse racial, dietary, and environmental backgrounds. Therefore, developing more precise animal models (e.g., humanized mice or organoid coculture systems) and integrating artificial intelligence (AI)-driven causal inference algorithms will be key to overcoming this bottleneck [299]. Traditional causality validation methods rely on animal models and in vitro experiments, which struggle to fully replicate the complex human immune environment. Future innovations in causal inference technologies are essential.

### Clinical heterogeneity in autoimmune patients and spatiotemporal heterogeneity of gut microbiota dynamics

The gut microbiota is a highly dynamic ecosystem, with its composition and function influenced by time, space, and environmental factors. This complexity is further amplified by the significant heterogeneity observed in autoimmune patients, which not only complicates the understanding of AID mechanisms but also raises the bar for designing effective intervention strategies. Future advancements in single-cell sequencing technologies for gut microbiota will enable the study of adaptive changes in individual bacteria and their interactions, offering critical insights for the precise diagnosis and treatment of AID [300]. AID progress through multiple stages, with abnormal changes in gut microbiota occurring even in the preclinical phase. These changes also exhibit seasonal variations, highlighting the temporal fluctuations in microbiota composition. Current research reveals that gut microbiota heterogeneity is not only spatial but also temporally dynamic, potentially reflecting shifts in the immune status of autoimmune patients. However, this temporal variability adds complexity to intervention strategies. In addition, regional environmental factors such as diet, antibiotic use, and lifestyle have been widely reported to influence gut microbiota. Nevertheless, the mechanisms underlying these environmental influences and their differential effects among individual autoimmune patients require further investigation.

### Limitations of gut-microbiota-based intervention strategies in treating AIDs

Although existing FMT therapies have shown efficacy in certain AID/RID, such as IBD and SLE, the heterogeneity of donor microbiota and variability in treatment efficacy (30% to 70% response rates) remain major challenges. In addition, safety concerns surrounding FMT persist, and issues such as genetic stability and colonization resistance in engineered bacteria require further optimization. The long-term stability and safety of engineered bacteria are major obstacles to their clinical translation. Furthermore, the colonization capacity of engineered bacteria is influenced by competition from the host microbiota and immune clearance, which may limit their therapeutic effectiveness. In terms of technical barriers in metabolite regulation, although metabolites such as SCFAs, bile acids, and tryptophan metabolites demonstrate immunomodulatory potential [301,302], their complex in vivo pharmacokinetics make precise delivery difficult. The clinical translation of such delivery systems requires interdisciplinary collaboration, integrating materials science, pharmaceutical chemistry, and biomedical engineering. For metabolite delivery systems, developing efficient delivery platforms is crucial for precise intervention. For example, hydrogel-based delivery systems can enable sustained release of SCFAs to intestinal inflammatory sites, but the biocompatibility and scalability of these systems require further optimization. Regarding data integration and analysis challenges, the advancement of multiomics technologies has led to increasing efforts to integrate genomic, transcriptomic, proteomic, and metabolomic data to comprehensively decipher microbiota–host interaction networks [303]. However, the heterogeneity and complexity of these data demand more sophisticated analytical methods.

Key obstacles to the clinical application of microbiota-based interventions for AID include the need for personalized treatment strategies that integrate host genomic data (e.g., HLA-DRB1*04 risk alleles), metabolomic profiles, and immune phenotypes. Currently, there is a lack of standardized multiomics analysis frameworks, and the development of clinical decision support systems lags behind. In addition, the impact of factors such as diet, lifestyle, and environmental exposures on microbiota intervention efficacy has not been fully evaluated, further complicating personalized treatment. Although personalized treatment is considered the future direction, its feasibility in clinical practice remains challenging. Even within the same patient population, individual responses to the same microbiota intervention strategy vary, highlighting the need for more refined stratification strategies to improve treatment precision. Regarding unknown long-term safety risks, while FMT and engineered bacteria therapies have shown short-term safety, their long-term effects remain unclear. For example, horizontal gene transfer in engineered bacteria may cause ecological disturbances, and phage therapy may lead to unpredictable microbiota imbalances. Therefore, establishing a comprehensive safety evaluation system is a prerequisite for advancing microbiota-based interventions into clinical practice. Long-term follow-up studies are essential to assess the safety of microbiota interventions in AIDs, emphasizing the importance of sustained monitoring. Regulatory and ethical issues also pose challenges, as rapid advancements in microbiota intervention technologies require regulatory agencies to develop unified standards.

### Future breakthrough directions

The first breakthrough lies in the innovation of precise microbiota editing technologies. CRISPR-based targeted editing and synthetic ecology strategies (e.g., constructing artificial microbiota consortia) hold promise for directed regulation of microbiota functions. By ingeniously modifying probiotics to create probiotic hybrid systems (PHSs), the biological behavior of probiotics in vivo can be regulated, enhancing their interaction with intestinal components and enabling advanced treatments for IBD. This is attributed to the intelligent response of PHS to the microenvironment and the smart design of multifunctional PHS combinations [304]. Future research should further optimize the stability and controllability of these technologies and explore their applicability across different disease contexts. In addition, combining microbiota interventions with traditional immunotherapies (e.g., Janus kinase inhibitors) may yield synergistic effects, as single intervention strategies often fall short of ideal efficacy. The integration of multimodal intervention strategies offers substantial advantages, and future studies should investigate optimal combinations and their mechanisms of action.

Leveraging AI technologies, the development of AI-driven personalized microbiota intervention prediction systems and digital twin models integrating microbiota dynamics, immune status, and clinical indicators can enable virtual validation of treatment plans, providing real-time decision support for clinical practice and greatly improving treatment precision and efficiency. Gut microbiota research spans multiple disciplines, including microbiology, immunology, metabolomics, and bioinformatics, and future breakthroughs will rely on interdisciplinary collaboration. For example, combining machine learning algorithms to mine large-scale multiomics data, developing novel delivery systems for precise metabolite release, and designing functional engineered bacteria using synthetic biology all require the collective efforts of multidisciplinary teams.

In terms of precision medicine and personalized interventions for gut microbiota in AIDs, engineered probiotics may emerge as novel treatments [297]. Key areas include (a) microbiome engineering therapies: developing gene-edited “smart probiotics” (e.g., CRISPR-modified strains) to degrade proinflammatory metabolites or deliver anti-inflammatory molecules and designing synthetic microbial communities (e.g., SynBio consortia) based on patient microbiota profiles for “Microbiota Transplantation 2.0”; (b) AI-driven therapy optimization: integrating multiomics data (metagenomics, metabolomics, and immunomics) to build digital twin models for predicting individualized microbiota interventions and using machine learning to map strain-drug interaction networks to avoid conflicts between antibiotics, immunosuppressants, and probiotics; (c) stratified treatment strategies: developing differentiated intervention plans based on AID subtypes (e.g., RA versus SLE) and genetic backgrounds (e.g., HLA genotypes).

In terms of technological innovation and multimodal integration for gut microbiota in AIDs, key areas include the following: (a) spatial omics technologies: using spatial transcriptomics and nanoscale mass spectrometry imaging to resolve 3-dimensional interaction maps of microbiota–host cells in intestinal mucosa; (b) dynamic monitoring systems: developing wearable gut biosensors to monitor real-time fluctuations in microbial metabolites (e.g., butyrate) and disease activity; (c) organoid and organ-on-chip models: constructing patient-derived gut organoid-immune cell coculture systems to simulate individualized responses to microbiota interventions.

Regarding clinical translation and industrialization challenges for gut microbiota in AIDs, key areas include the following: (a) standardization and regulatory frameworks: establishing production standards for microbiota-based drugs (e.g., stability and dosage control for live biotherapeutics) and promoting guidelines for microbiome therapies by regulatory agencies such as the US Food and Drug Administration (FDA) and European Medicines Agency (EMA); (b) clinical trial design innovation: adopting adaptive clinical trial platforms to dynamically adjust microbiota interventions (e.g., FMT combined with immunosuppressants) and exploring microbiome end-point indicators (e.g., microbiota diversity indices and metabolite thresholds) as alternatives to traditional clinical scores; (c) long-term safety tracking: launching a global microbiome safety database to monitor long-term risks of microbiota interventions (e.g., secondary diseases caused by microbiota imbalances).

In terms of multidisciplinary collaboration in public health and preventive medicine for gut microbiota in AIDs, key areas include the following: (a) early intervention windows: conducting neonatal microbiome screening in genetically high-risk populations and shaping antiautoimmune microbiota through human milk oligosaccharides and early probiotics; (b) diet–microbiota interaction guidelines: developing anti-inflammatory dietary frameworks based on regional diets (e.g., Mediterranean versus Asian diets) and specifying doses of dietary fiber and polyphenols for microbiota modulation; (c) environmental exposure management: studying the impact of antibiotic overuse and pesticide residues on microbiota–AID associations in urbanized settings and advocating for policy-level microbiome protection initiatives.

**Fig. 7.**
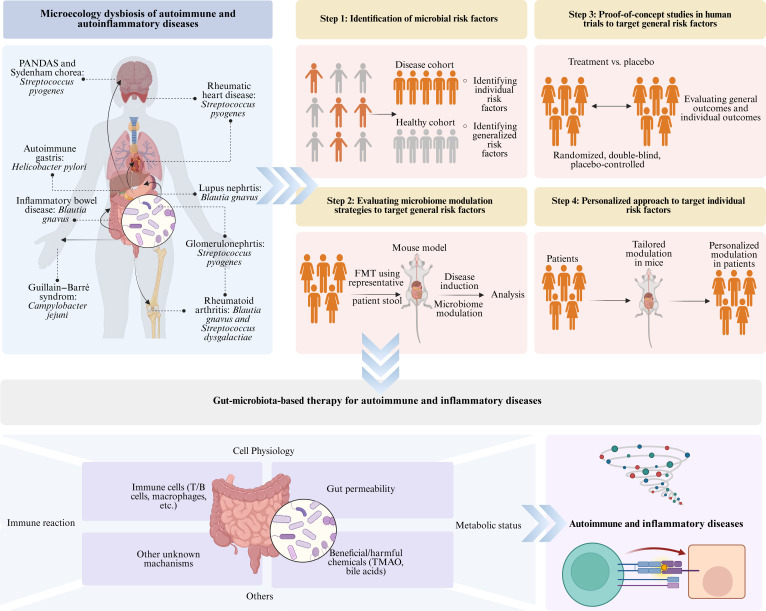
Future perspectives on gut-microbiota-based therapies in AIDs. AIDs are associated with alterations in gut microbiota, driven by mechanisms such as immune cell dysregulation, changes in gut permeability, imbalances in beneficial and harmful microbial metabolites, and other unknown factors. This figure proposes a study design for developing microbiota modulation strategies in patients with lupus: (a) Cross-sectional and longitudinal comparisons between lupus patients and healthy individuals identify overarching and individual risk factors; (b) overarching risk factors are validated using FMT into GF mice, followed by the development of modulation strategies (e.g., dietary interventions); (c) modulation strategies are evaluated in placebo-controlled, randomized, double-blind human trials, with individual responses guiding further optimization; (d) Individualized strategies are tested in GF mice transplanted with patient-specific stool samples, with successful approaches applied to treat corresponding patients. Microbiota-based therapies target cell physiology, metabolic status, immune responses, and other mechanisms to restore microbial balance and improve disease outcomes.TMAO, trimethylamine *N*-oxide. This figure was created using Biorender.com.

Finally, we hope that global collaboration among researchers can achieve microbiota-immune system reprogramming for AIDs, increasing disease remission rates from 30% to over 70%. Breakthroughs in biomarkers include advancements in microbiome vaccines (e.g., mucosal vaccines targeting *P. copri*) and closed-loop regulatory systems (e.g., implantable devices releasing prebiotics/postbiotics based on real-time microbiota data). Interdisciplinary integration will reveal shared mechanisms between AIDs, cancer, and depression, advancing our understanding of comorbid conditions (Fig. 7).
