# Supplementary material for: The Gut Microbiota: Emerging Evidence in Autoimmune and Inflammatory Diseases
Source: Research (Wash D C). 2026 Feb 4;9:1097. doi: 10.34133/research.1097 (PMC12868559; doi:10.34133/research.1097)
Supplement: Supplementary 1 — Text S1 and S2 Figs. S1 and S2 Tables S1 to S4 [file research.1097.f1.zip › Supplementary Material Text S1.docx]

**Supplementary Material-Text S1**

1. **Involvement of Gut Microbiota Metabolites in Immune System Regulation**

The gut microbiota generates various metabolites through different metabolic pathways. Metabolomics is a crucial technique for a profound understanding of gut microbial ecology, primarily acquiring data through mass spectrometry (MS) or nuclear magnetic resonance (NMR) methodologies[1-2]. In this section, we delve into how gut microbial metabolites intricately participate in intestinal immune system regulation. Among all the bacterial metabolites, short-chain fatty acids, amino acid derivatives, and secondary bile acids typically stem from the gut microbial community, whereas lactic acid and succinic acid are synthesized jointly by humans and microbes[3]. Irrespective of their origins, these metabolites play critical roles in the differentiation and functioning of innate and adaptive immune cells within the gut, directly or indirectly influencing the host's immune responses, promoting or impeding the onset of autoimmune diseases. The predominant metabolites under extensive study include short-chain fatty acids (SCFAs), tryptophan and its derivatives, and bile acids[3].

**1.1 SCFAs**

**1.1.1 Formation Pathway**

SCFAs mainly originate from dietary fibers and colon mucus layers rich in heavily O-glycosylated mucins. Bacteria involved in their formation include Bacteroides, Lactobacillus, Bifidobacterium, and Clostridium[4]. Complex carbohydrates in the dietary fiber or mucins are initially hydrolyzed by microbes into pentose or hexose monosaccharides, metabolized through either the pentose phosphate pathway (pentose) or the Embden-Meyerhof-Parnas pathway (hexose) into pyruvic acid. Pyruvic acid then undergoes a series of biochemical reactions to eventually form SCFAs, with acetate, propionate, and butyrate constituting over 95% of the total SCFAs.

**1.1.2 Function/Mechanism**

As one of the most abundant microbial metabolites in the gut, SCFAs can impact various host physiological functions. In particular, attention has been drawn to their role in regulating host immune function. In terms of innate immunity, researchers have found that SCFAs can inhibit dendritic cell (DC) maturation, influence the secretion of the cytokine interleukin (IL)-23[5], and modulate macrophage function and cytokine secretion[6]. In the context of adaptive immunity, Furusawa et al. [7] and Smith et al. [8] explored the mechanisms through which SCFAs influence immune cell behavior, focusing on their ability to regulate colonic regulatory T (Treg) cells, promote IgA production in the intestines [9], enhance the secretion of anti-inflammatory IL-10 by Th1 cells [10], boost the memory potential of antigen-activated CD8+ T cells [11], and induce IL-22 production by CD4+ T cells [12]. These findings demonstrate that SCFAs contribute to intestinal immune homeostasis by modulating Treg cell populations and functions, enhancing IgA production, promoting anti-inflammatory cytokine secretion, and supporting T cell memory and function, underscoring their potential therapeutic relevance in immune-related intestinal conditions.. SCFAs primarily regulate host cell functions through the following three mechanisms: (1) Serving as carbon sources for host endogenous metabolic products. In the SCFAs family, butyrate is utilized to supply energy for oxidative phosphorylation through sustained fatty acid metabolism, thereby extending the tricarboxylic acid cycle beyond glycolytic metabolites and enhancing cellular energy metabolism. Additionally, it promotes the memory potential of activated CD8+ T cells. [11]. (2) Acting as ligands to activate host G protein-coupled receptors (GPCRs, primarily including GPR43, GPR41, and GRP109A) signaling pathways. For instance, SCFAs can promote the secretion of IL-10 by Th1 cells by activating GPR43[10]. (3) Inhibiting histone deacetylases (HDAC) impacting host gene expression[13−14]. SCFAs have been demonstrated to stimulate the generation and augment the functionality of Treg cells. The primary mechanisms involve the inhibition of histone deacetylase (HDAC) to enhance histone H3 acetylation, thereby upregulating FOXP3 expression, as well as the activation of the GPR43 receptor, which triggers Treg cell proliferation via IL-10 secretion [7−8, 13]. Furthermore, SCFAs can elicit IL-22 production from CD4+ T cells and innate lymphoid cells by engaging the GPR41 receptor on the cell membrane and suppressing HDAC activity, which facilitates aryl hydrocarbon receptor (AhR) and hypoxia-inducible factor 1α (HIF1α) expressions[12].

**1.1.3 Short-Chain Fatty Acids (SCFAs) and Autoimmune Diseases**

Short-chain fatty acids (SCFAs) have been implicated in the pathogenesis of multiple autoimmune disorders, including RA, SLE, MS, pSS, and IBD. Studies have shown that propionic acid levels are decreased in the fecal samples of RA patients and RA model mice, and supplementation with propionic acid can alleviate arthritis symptoms by activating AhR to regulate Breg cell function[14]. SCFAs can alleviate the severity of various RA model mice, including collagen-induced arthritis (CIA)[65] and antigen-induced arthritis (AIA)[14]. It has been found that, in the fecal samples of SLE patients, SCFA levels are related to the gut microbiota structure[16]. The inclusion of resistant starch or SCFAs in the diet of Toll-like receptor 7-dependent SLE mouse models has been demonstrated to suppress the proliferation of *Lactobacillus reuteri* and mitigate disease phenotypes. [17]. However, whether SCFAs have a protective effect on SLE patients requires further basic and clinical research for validation. In recent years, the regulation of the microbiota-gut-brain axis has gained increasing attention in the pathogenesis of neurological autoimmune diseases like MS. Various gut microbial strains that produce SCFAs have been found to decrease in MS patients[18]. Treating experimental autoimmune encephalomyelitis (EAE) model mice with propionic acid can increase the local intestinal Treg cell content, which exerts anti-inflammatory effects and effectively alleviates disease symptoms[18]. Besides affecting the intestinal region, the effects of SCFAs can extend to the central nervous system by regulating the maturation and function of microglial cells in the central nervous system through the GPR43 receptor[19]. In pSS patients, microbial strains producing butyric acid are also decreased[20]. Studies have demonstrated that butyric acid, synthesized by intestinal microbiota, can exert its anti-inflammatory properties not only within the gastrointestinal tract but also in ocular tissues, primarily through the modulation of Treg cells. [21]. Further in-depth studies are needed to explore whether similar mechanisms exist in pSS using animal models. Fecal metabolomics studies have revealed that the levels of SCFAs in the intestines of IBD patients are lower than those in healthy control groups[22], and in T cell-dependent chronic colitis models and dextran sulfate sodium (DSS)-induced colitis models, SCFAs can alleviate the severity of colitis[57,60]. Additionally, SCFA receptor knockout mice exhibit susceptibility to colitis, further demonstrating the protective effects of SCFAs in IBD[23].

**1.2 Tryptophan and Its Derivatives**

**1.2.1 Formation Pathways**

Approximately 5% to 10% of dietary proteins escape absorption in the small intestine and reach the colon, where they are metabolized by the gut microbiota into amino acids and fermentation byproducts such as amines, phenols, indoles, and sulfur-containing compounds. Tryptophan, an essential amino acid, is a key substrate in multiple metabolic pathways, producing signaling molecules like vitamin B3, serotonin, indole, and nicotinamide adenine dinucleotide (NAD). Its metabolic fate is shaped by the gut microbiota through several pathways: the indole pathway (yielding indole, indoxyl sulfate, NAD, etc.), the serotonin pathway (producing serotonin), protein synthesis pathways, and direct conversions (generating tryptamine, indole, etc.), each resulting in distinct metabolites[24].

**1.2.2 Functions/Mechanisms**

Tryptophan and its derivatives primarily regulate intestinal and host systemic immune functions by acting on the pregnane X receptor (PXR) or AhR. AhR is ubiquitously expressed across immune cells and serves as a critical regulator of immune responses, particularly in modulating CD4+ T cells. Indole and its derivatives regulate T cell differentiation, suppressing Th17 cells while promoting Treg cell generation[25]. Additionally, indole derivatives produced by *Lactobacillus reuteri* facilitate the conversion of CD4+ T cells into CD4+CD8+ double-positive intraepithelial lymphocytes, maintaining intestinal immune homeostasis[26].

**1.2.3 Tryptophan and Its Derivatives in Autoimmune Diseases**

Current research indicates a close association of tryptophan metabolites with diseases such as RA, MS, and IBD. Pongratz et al.[27] found correlations between serum tryptophan levels and its metabolites in RA patients with disease activity and rheumatoid factors. Indole-3-methanol can alleviate adjuvant-induced arthritis symptoms[28], while indole-3-aldehyde and indole-3-acetic acid can promote the secretion of cytokines by innate immune cells[29], suggesting that indole and its derivatives may have protective effects in RA. Tryptophan and its derivatives also play a role in the regulation of the gut-brain axis and are closely associated with MS. Circulating AhR agonist levels are reduced in MS patients, and supplementing MS model mice with tryptophan metabolites such as indole, 3-indoxyl sulfate, 3-indole propionic acid, and indole-3-aldehyde can alleviate central nervous system inflammation in EAE mice[30]. In IBD patients, the levels of tryptophan and indole acetic acid in feces are reduced, while indole levels are elevated[31]. The expression of the AhR receptor in the intestinal tissue of IBD patients is increased, whereas AhR activity in the stool is decreased[31]. In the DSS-induced colitis animal model, AhR-/- mice exhibit more severe intestinal inflammation, which can be significantly alleviated after treatment with AhR agonists[32].

**1.3 Bile Acids**

**1.3.1 Biosynthesis Pathways**

Bile acids, synthesized from cholesterol in the liver, solubilize dietary lipids and fat-soluble fibers in the small intestine. Their synthesis involves over 17 enzymes and occurs via two pathways: the classic pathway (75%, producing chenodeoxycholic acid [CDCA] and cholic acid [CA]) and the alternative pathway (predominantly yielding CDCA). While 95% of primary bile acids are reabsorbed in the ileum and recirculate to the liver, 5% reach the large intestine. There, gut microbiota transform primary bile acids into secondary bile acids through enzymatic processes like dehydroxylation, hydroxylation isomerization, and oxidation. The most abundant secondary bile acids are deoxycholic acid (DCA) and lithocholic acid (LCA), though the precise molecular mechanisms remain under investigation[33].

**1.3.2 Functions/Mechanisms**

Bile acids exert their physiological effects primarily by activating the farnesoid X receptor (FXR) and G protein-coupled bile acid receptor 1 (GPBAR1/TGR5). Unconjugated bile acids also bind to nuclear receptors such as the pregnane X receptor (PXR), constitutive androstane receptor (CAR), and vitamin D receptor (VDR)[34]. In innate immunity, secondary bile acids like deoxycholic acid (DCA) stimulate Kupffer cells to produce reactive oxygen species (ROS), while primary and secondary bile acids suppress pro-inflammatory responses in macrophages via TGR5 and PKA pathways[35]. The TGR5 agonist BAR501 promotes macrophage polarization from the M1 to M2 phenotype[23]. In adaptive immunity, bile acid derivatives play emerging roles: IsoalloLCA enhances Treg cell differentiation by acetylating the CNS3 element of FOXP3, while 3-oxoLCA inhibits Th17 cell differentiation by interacting with RORγt[36]. In the gut, bile acids activate vitamin D receptors, driving the generation of RORγa+ regulatory T cells to maintain mucosal immune balance. Notably, in liver cancer, primary bile acids promote, while secondary bile acids inhibit, natural killer T (NKT) cell accumulation, highlighting their context-dependent roles[37]. Further research is needed to explore these mechanisms in other conditions, potentially revealing new therapeutic targets for immune-related diseases[38].

**1.3.3 Bile Acids and Autoimmune Diseases**

Current research underscores the significant involvement of bile acid metabolites in the pathogenesis of multiple systemic and organ-specific disorders, including PBC, RA, SLE, and IBD[39-45]. These metabolites serve as critical regulators of the gut-liver axis, orchestrating essential physiological processes that maintain inter-organ homeostasis.

Bile stasis is a significant clinical manifestation of PBC, and early administration of the bile acid sequestrant ursodeoxycholic acid (UDCA) in PBC patients has shown promising therapeutic effects. Studies have revealed abnormalities in bile acid profiles in both feces and serum of PBC patients, which is correlated with disease severity[39]. Treatment with UDCA leads to a significant decrease in bile acid circulation in PBC patients, accompanied by alterations in gut microbiota composition[40].

Recent investigations have identified dysregulated bile acid metabolism in RA patients, with abnormalities observed in both serum and synovial fluid profiles[41]. Mechanistically, the secondary bile acid lithocholic acid (LCA) exerts anti-inflammatory properties through TGR5 receptor activation, as demonstrated in collagen-induced arthritis (CIA) murine models[42]. Meagan et al.[46] identified at-risk individuals based on serum autoantibodies present years before clinical RA diagnosis, further isolating specific bacteria labeled by these antibodies through fecal analysis. Animal models confirmed these bacteria trigger both autoantibody production and full disease development. He et al.[47] revealed that butyrate-producing bacteria influence colonic butyrate levels, impacting RA activity, antibody production, and joint deformation, highlighting butyrate’s immune-regulatory mechanisms and therapeutic potential. Balakrishnan’s study[48] demonstrated that *Eggerthella lenta* exacerbates RA severity in genetically susceptible mice by increasing rheumatoid factors, pro-inflammatory cytokines, and follicular T helper cells while reducing regulatory T cells and amino acid levels, with gender-specific effects observed. Luo et al.[49] linked gut microbiota alterations in high-risk individuals to metabolic disruptions and mucosal immune imbalances, triggering arthritis. Hong et al.[50] further elucidated the gut-bone axis, showing that *Fusobacterium nucleatum*-derived outer membrane vesicles target the Rab5a-YB-1 axis, delivering FadA-containing proteins to joints and exacerbating inflammation. These findings collectively underscore the complex interplay between gut microbiota, immune responses, and RA pathogenesis, suggesting that microbial dysbiosis, specific bacterial metabolites, and bile acid metabolites may serve as both biomarkers and therapeutic targets in RA management, although their precise mechanistic contributions to disease pathogenesis require further elucidation.

Comprehensive lipidomic analyses of serum and fecal samples from SLE patients have also revealed distinct alterations in bile acid metabolic profiles, showing significant correlations with disease activity indices. Notably, SLE patients demonstrate increased fecal concentrations of primary bile acids, including cholic acid, glycocholic acid, and taurocholic acid, compared to healthy controls[43]. Downregulation of FXR receptor expression was observed in both SLE patients and MRL/lpr lupus-prone mice. Activation of the FXR receptor suppressed the secretion of inflammatory factors in lupus-prone mice, alleviating liver damage[44].

Studies have further elucidated the role of bile acids in the pathophysiology of IBD as well. In DSS-treated mice, the mixture of primary and secondary bile acids can relieve DSS-induced colitis[37]. Various bile acid receptors play a role in regulating the onset of colitis. For instance, the FXR receptor agonist INT-747 mitigates DSS-induced colitis, while FXR-deficient mice show exacerbated colitis[45]. TGR5 receptor agonist BAR501 exhibits protective effects in a T cell-mediated colitis animal model[23]. These studies collectively suggest the involvement of bile acid metabolites in IBD pathogenesis. However, further research is needed to clarify the precise mechanisms and explore translational applications, particularly in early intervention and personalized treatment strategies.

**References:**

1 Loh JS, Mak WQ, Tan LKS, Ng CX, Chan HH, Yeow SH, Foo JB, Ong YS, How CW, Khaw KY. Microbiota-gut-brain axis and its therapeutic applications in neurodegenerative diseases. Signal Transduct Target Ther. 2024;9(1):37. doi: 10.1038/s41392-024-01743-1.

2. Campbell C, Kandalgaonkar MR, Golonka RM, Yeoh BS, Vijay-Kumar M, Saha P. Crosstalk between Gut Microbiota and Host Immunity: Impact on Inflammation and Immunotherapy. Biomedicines. 2023;11(2):294. doi: 10.3390/biomedicines11020294.

3. Takeuchi T, Nakanishi Y, Ohno H. Microbial Metabolites and Gut Immunology. Annu Rev Immunol. 2024;42(1):153-178. doi: 10.1146/annurev-immunol-090222-102035.

4. Schroeder BO. Fight them or feed them: how the intestinal mucus layer manages the gut microbiota. Gastroenterol Rep (Oxf). 2019;7(1):3-12. doi: 10.1093/gastro/goy042.

5. Nastasi C, Candela M, Bonefeld CM, Geisler C, Hansen M, Krejsgaard T, Biagi E, Andersen MH, Brigidi P, Ødum N, Litman T, Woetmann A. The effect of short-chain fatty acids on human monocyte-derived dendritic cells. Sci Rep. 2015;5:16148. doi: 10.1038/srep16148.

6. Schulthess J, Pandey S, Capitani M. The Short Chain Fatty Acid Butyrate Imprints an Antimicrobial Program in Macrophages. Immunity, 2019, 50: 432-445. e7.

7. Furusawa Y, Obata Y, Fukuda S, Endo TA, Nakato G, Takahashi D, Nakanishi Y, Uetake C, Kato K, Kato T, Takahashi M, Fukuda NN, Murakami S, Miyauchi E, Hino S, Atarashi K, Onawa S, Fujimura Y, Lockett T, Clarke JM, Topping DL, Tomita M, Hori S, Ohara O, Morita T, Koseki H, Kikuchi J, Honda K, Hase K, Ohno H. Commensal microbe-derived butyrate induces the differentiation of colonic regulatory T cells. Nature. 2013;504(7480):446-50. doi: 10.1038/nature12721.

8. Smith PM, Howitt MR, Panikov N, Michaud M, Gallini CA, Bohlooly-Y M, Glickman JN, Garrett WS. The microbial metabolites, short-chain fatty acids, regulate colonic Treg cell homeostasis. Science. 2013;341(6145):569-73. doi: 10.1126/science.1241165.

9. Wu W, Sun M, Chen F, Cao AT, Liu H, Zhao Y, Huang X, Xiao Y, Yao S, Zhao Q, Liu Z, Cong Y. Microbiota metabolite short-chain fatty acid acetate promotes intestinal IgA response to microbiota which is mediated by GPR43. Mucosal Immunol. 2017;10(4):946-956. doi: 10.1038/mi.2016.114.

10. Sun M, Wu W, Chen L, Yang W, Huang X, Ma C, Chen F, Xiao Y, Zhao Y, Ma C, Yao S, Carpio VH, Dann SM, Zhao Q, Liu Z, Cong Y. Microbiota-derived short-chain fatty acids promote Th1 cell IL-10 production to maintain intestinal homeostasis. Nat Commun. 2018;9(1):3555. doi: 10.1038/s41467-018-05901-2.

11. Bachem A, Makhlouf C, Binger KJ. Microbiota Derived Short-Chain Fatty Acids Promote the Memory Potential of Antigen-Activated CD8(+) T Cells. Immunity, 2019, 51: 285-297. e5.

12. Yang W, Yu T, Huang X. Intestinal microbiota-derived short-chain fatty acids regulation of immune cell IL-22 production and gut immunity. Nat Commun, 2020, 11: 4457.

13. Atarashi K, Tanoue T, Oshima K. Treg induction by a rationally selected mixture of Clostridia strains from the human microbiota. Nature, 2013, 500: 232-236.

14. Rosser EC, Piper CJM, Matei DE. Microbiota Derived Metabolites Suppress Arthritis by Amplifying Aryl Hydrocarbon Receptor Activation in Regulatory B Cells. Cell Metab, 2020, 31: 837-851. e10.

15. Mizuno M, Noto D, Kaga N, Chiba A, Miyake S. The dual role of short fatty acid chains in the pathogenesis of autoimmune disease models. PLoS One. 2017;12(2):e0173032. doi: 10.1371/journal.pone.0173032.

16. Rodriguez-Carrio J, Lopez P, Sanchez B. Intestinal Dysbiosis Is Associated with Altered Short-Chain Fatty Acids and Serum-Free Fatty Acids in Systemic Lupus Erythematosus. Front Immunol, 2017, 8: 23.

17. Zegarra-Ruiz DF, El Beidaq A, Iñiguez AJ, Lubrano Di Ricco M, Manfredo Vieira S, Ruff WE, Mubiru D, Fine RL, Sterpka J, Greiling TM, Dehner C, Kriegel MA. A Diet-Sensitive Commensal Lactobacillus Strain Mediates TLR7-Dependent Systemic Autoimmunity. Cell Host Microbe. 2019;25(1):113-127.e6. doi: 10.1016/j.chom.2018.11.009.

18. Haghikia A, Jörg S, Duscha A, Berg J, Manzel A, Waschbisch A, Hammer A, Lee DH, May C, Wilck N, Balogh A, Ostermann AI, Schebb NH, Akkad DA, Grohme DA, Kleinewietfeld M, Kempa S, Thöne J, Demir S, Müller DN, Gold R, Linker RA. Dietary Fatty Acids Directly Impact Central Nervous System Autoimmunity via the Small Intestine. Immunity. 2015;43(4):817-29. doi: 10.1016/j.immuni.2015.09.007.

19. Erny D, Hrabě de Angelis AL, Jaitin D, Wieghofer P, Staszewski O, David E, Keren-Shaul H, Mahlakoiv T, Jakobshagen K, Buch T, Schwierzeck V, Utermöhlen O, Chun E, Garrett WS, McCoy KD, Diefenbach A, Staeheli P, Stecher B, Amit I, Prinz M. Host microbiota constantly control maturation and function of microglia in the CNS. Nat Neurosci. 2015;18(7):965-77. doi: 10.1038/nn.4030.

20. Cano-Ortiz A, Laborda-Illanes A, Plaza-Andrades I, Membrillo Del Pozo A, Villarrubia Cuadrado A, Rodríguez Calvo de Mora M, Leiva-Gea I, Sanchez-Alcoholado L, Queipo-Ortuño MI. Connection between the Gut Microbiome, Systemic Inflammation, Gut Permeability and FOXP3 Expression in Patients with Primary Sjögren's Syndrome. Int J Mol Sci. 2020;21(22):8733. doi: 10.3390/ijms21228733.

21. Chen X, Su W, Wan T. Sodium butyrate regulates Th17 /Treg cell balance to ameliorate uveitis via the Nrf2 /HO- 1 pathway. Biochem Pharmacol, 2017, 142: 111-119.

22. Lloyd-Price J, Arze C, Ananthakrishnan AN, Schirmer M, Avila-Pacheco J, Poon TW, Andrews E, Ajami NJ, Bonham KS, Brislawn CJ, Casero D, Courtney H, Gonzalez A, Graeber TG, Hall AB, Lake K, Landers CJ, Mallick H, Plichta DR, Prasad M, Rahnavard G, Sauk J, Shungin D, Vázquez-Baeza Y, White RA 3rd; IBDMDB Investigators; Braun J, Denson LA, Jansson JK, Knight R, Kugathasan S, McGovern DPB, Petrosino JF, Stappenbeck TS, Winter HS, Clish CB, Franzosa EA, Vlamakis H, Xavier RJ, Huttenhower C. Multi-omics of the gut microbial ecosystem in inflammatory bowel diseases. Nature. 2019;569(7758):655-662.doi: 10.1038/s41586-019-1237-9.

23. Biagioli M, Carino A, Cipriani S, Francisci D, Marchianò S, Scarpelli P, Sorcini D, Zampella A, Fiorucci S. The Bile Acid Receptor GPBAR1 Regulates the M1/M2 Phenotype of Intestinal Macrophages and Activation of GPBAR1 Rescues Mice from Murine Colitis. J Immunol. 2017 ;199(2):718-733.doi: 10.4049/jimmunol.1700183.

24. Krautkramer KA, Fan J, Backhed F. Gut microbial metabo lites as multi-kingdom intermediates. Nat Rev Microbiol, 2021, 19: 77-94.

25. Singh NP, Singh UP, Rouse M, Zhang J, Chatterjee S, Nagarkatti PS, Nagarkatti M. Dietary Indoles Suppress Delayed-Type Hypersensitivity by Inducing a Switch from Proinflammatory Th17 Cells to Anti-Inflammatory Regulatory T Cells through Regulation of MicroRNA. J Immunol. 2016 Feb 1;196(3):1108-22.doi: 10.4049/jimmunol.1501727.

26. Cervantes-Barragan L, Chai JN, Tianero MD. Lacto bacillus reuteri induces gut intraepithelial CD4 (+) CD8 alphaalpha(+) T cells. Science, 2017, 357: 806-810.

27. Pongratz G, Lowin T, Sewerin P. Tryptophan metabo lism in rheumatoid arthritis is associated with rheumatoid fac tor and predicts joint pathology evaluated by the Rheumatoid Arthritis MRI Score(RAMRIS). Clin Exp Rheumatol, 2019, 37: 450-457.

28. Hasan H, Ismail H, El-Orfali Y. Therapeutic benefits of Indole- 3-Carbinol in adjuvant-induced arthritis and its protective effect against methotrexate induced-hepatic toxicity. BMC Complement Altern Med, 2018, 18: 337.

29. Langan D, Perkins DJ, Vogel SN. Microbiota-Derived Metabolites, Indole- 3-aldehyde and Indole- 3-acetic Acid, Differentially Modulate Innate Cytokines and Stromal Remod eling Processes Associated with Autoimmune Arthritis. Int J Mol Sci, 2021, 22.

30. Rothhammer V, Mascanfroni ID, Bunse L. Type I in terferons and microbial metabolites of tryptophan modulate astrocyte activity and central nervous system inflammation via the aryl hydrocarbon receptor. Nat Med, 2016, 22: 586-597.

31. Lamas B, Richard ML, Leducq V. CARD9 impacts colitis by altering gut microbiota metabolism of tryptophan into aryl hydrocarbon receptor ligands. Nat Med, 2016, 22: 598-605.

32. Stockinger B, Shah K, Wincent E. AHR in the intestinal microenvironment: safeguarding barrier function. Nat Rev Gastroenterol Hepatol, 2021, 18: 559-570.

33. Funabashi M, Grove TL, Wang M. A metabolic pathway for bile acid dehydroxylation by the gut microbiome. Nature, 2020, 582: 566-570.

34. Jia W, Xie G, Jia W. Bile acid-microbiota crosstalk in gastrointestinal inflammation and carcinogenesis. Nat Rev Gastroenterol Hepatol, 2018, 15: 111-128.

35. Fiorucci S, Biagioli M, Zampella A. Bile Acids Acti vated Receptors Regulate Innate Immunity. Front Immunol, 2018, 9: 1853.

36. Hang S, Paik D, Yao L. Bile acid metabolites control TH17 and Treg cell differentiation. Nature, 2019, 576: 143-148.

37. Song X, Sun X, Oh SF. Microbial bile acid metabolites modulate gut RORgamma(+) regulatory T cell homeostasis. Nature, 2020, 577: 410-415.

38. Ma C, Han M, Heinrich B. Gut microbiome-mediated bile acid metabolism regulates liver cancer via NKT cells. Science, 2018, 360: eaan5931.

39. Chen W, Wei Y, Xiong A. Comprehensive Analysis of Serum and Fecal Bile Acid Profiles and Interaction with Gut Microbiota in Primary Biliary Cholangitis. Clin Rev Allergy Immunol, 2020, 58: 25-38.

40. Li B, Zhang J, Chen Y. Alterations in microbiota and their metabolites are associated with beneficial effects of bile acid sequestrant on icteric primary biliary Cholangitis. Gut Microbes, 2021, 13: 1946366.

41. Bartikoski BJ, De Oliveira MS, Do Espirito Santo RC. A Review of Metabolomic Profiling in Rheumatoid Arthritis: Bringing New Insights in Disease Pathogenesis, Treatment and Comorbidities. Metabolites, 2022, 12: 394.

42. Li ZY, Zhou JJ, Luo CL. Activation of TGR5 alleviates inflammation in rheumatoid arthritis peripheral blood mononuclear cells and in mice with collagen II-induced arthritis. Mol Med Rep, 2019, 20: 4540-4550.

43. He J, Chan T, Hong X. Microbiome and Metabolome Analyses Reveal the Disruption of Lipid Metabolism in Systemic Lupus Erythematosus. Front Immunol, 2020, 11: 1703.

44. Lian F, Wang Y, Chen J. Activation of farnesoid X receptor attenuates liver injury in systemic lupus erythematosus. Rheumatol Int, 2012, 32: 1705-1710.

45. Gadaleta RM, Garcia-Irigoyen O, Cariello M. Fibroblast Growth Factor 19 modulates intestinal microbiota and inflammation in presence of Farnesoid X Receptor. EBioMedicine, 2020, 54: 102719.

46. Chriswell ME, Lefferts AR, Clay MR, Hsu AR, Seifert J, Feser ML, Rims C, Bloom MS, Bemis EA, Liu S, Maerz MD, Frank DN, Demoruelle MK, Deane KD, James EA, Buckner JH, Robinson WH, Holers VM, Kuhn KA. Clonal IgA and IgG autoantibodies from individuals at risk for rheumatoid arthritis identify an arthritogenic strain of Subdoligranulum. Sci Transl Med. 2022;14(668):eabn5166.doi: 10.1126/scitranslmed.abn5166.

47. He J, Chu Y, Li J, Meng Q, Liu Y, Jin J, Wang Y, Wang J, Huang B, Shi L, Shi X, Tian J, Zhufeng Y, Feng R, Xiao W, Gan Y, Guo J, Shao C, Su Y, Hu F, Sun X, Yu J, Kang Y, Li Z. Intestinal butyrate-metabolizing species contribute to autoantibody production and bone erosion in rheumatoid arthritis. Sci Adv. 2022;8(6):eabm1511.doi: 10.1126/sciadv.abm1511.

48. Balakrishnan B, Luckey D, Wright K, Davis JM, Chen J, Taneja V. Eggerthella lenta augments preclinical autoantibody production and metabolic shift mimicking senescence in arthritis. Sci Adv. 2023 ;9(35):eadg1129.doi: 10.1126/sciadv.adg1129.

49. Luo Y, Tong Y, Wu L, Niu H, Li Y, Su LC, Wu Y, Bozec A, Zaiss MM, Qing P, Zhao H, Tan C, Zhang Q, Zhao Y, Tang H, Liu Y. Alteration of Gut Microbiota in Individuals at High-Risk for Rheumatoid Arthritis Associated With Disturbed Metabolome and the Initiation of Arthritis Through the Triggering of Mucosal Immunity Imbalance. Arthritis Rheumatol. 2023 ;75(10):1736-1748.doi: 10.1002/art.42616.

50. Hong M, Li Z, Liu H, Zheng S, Zhang F, Zhu J, Shi H, Ye H, Chou Z, Gao L, Diao J, Zhang Y, Zhang D, Chen S, Zhou H, Li J. Fusobacterium nucleatum aggravates rheumatoid arthritis through FadA-containing outer membrane vesicles. Cell Host Microbe. 2023;31(5):798-810.e7.doi: 10.1016/j.chom.2023.03.018.
