# Supplementary material for: The Gut Microbiota: Emerging Evidence in Autoimmune and Inflammatory Diseases
Source: Research (Wash D C). 2026 Feb 4;9:1097. doi: 10.34133/research.1097 (PMC12868559; doi:10.34133/research.1097)
Supplement: Supplementary 1 — Text S1 and S2 Figs. S1 and S2 Tables S1 to S4 [file research.1097.f1.zip › Supplementary Material Text S2.docx]

**2 Mechanism of Gut Microbiota in Multiple Sclerosis**

Multiple sclerosis (MS), a common inflammatory demyelinating disease of the central nervous system, is characterized by inflammatory plaques in the brain and spinal cord, leading to myelin and oligodendrocyte destruction and neuronal loss [1]. Gut microbiota plays a key role in MS development, as shown by animal studies and Next-Generation Sequencing (NGS) research. MS patients exhibit reduced abundance of beneficial bacteria like Clostridium clusters XIVa and IV, which produce butyrate and propionate, while pro-inflammatory genera increase [2–3]. Common features include decreased Prevotella and Parabacteroides and increased Akkermansia, which may promote TH1/TH17 differentiation and inflammation, though its exact role remains unclear [4]. A large multicenter study by the International Multiple Sclerosis Microbiome Study (iMSMS) team revealed significant microbial changes in MS patients, including increased Akkermansia muciniphila and decreased Prevotella copri, along with altered metabolic pathways [5].

In a recent study [6], the research team investigated the microbiota and metabolites in patients with stable multiple sclerosis, those with worsening conditions, and those who transitioned from relapsing-remitting to progressive multiple sclerosis within two years. The team found that short-chain fatty acid-producing microbes, such as Eubacterium hallii, Butyricoccaceae, Blautia, and other SCFA-producing microorganisms, were beneficially associated with improvements in disability levels, MRI metrics, cognitive function, and quality of life, whereas Alistipes showed harmful associations. Global metabolomics identified changes in serum and fecal metabolites in patients with progressive multiple sclerosis and in those who transitioned from relapsing to progressive disease. Most fecal metabolites linked to disease progression were reduced, indicating a deficiency in protective factors within the gut. In summary, gut microbiota were associated with changes in disability levels, MRI outcomes, and quality of life in multiple sclerosis patients over two years; serum and fecal metabolites were linked to the development of progressive multiple sclerosis; and patients with worsening conditions lacked potentially beneficial microbial metabolites.

Recent research also highlights sex-specific mechanisms, with dopamine D2 receptor activation in gut epithelial cells altering microbiota and metabolites in female mice, promoting MS onset [7]. These findings underscore the gut microbiota's role in MS and suggest potential gender-specific interventions.

- 1. **Microbiota-Gut-Brain Axis**

The microbiota-gut-brain axis represents a bidirectional communication pathway linking the brain and gastrointestinal tract, facilitated by the neuroendocrine system [8–9]. Comprising the central nervous system, autonomic nervous system, and enteric nervous system, it orchestrates neuroimmune and endocrine regulation. Collectively, elements alongside this axis coordinate the physiological activities of the gastrointestinal tract and the brain. Gut microbiota affects neural and gastrointestinal functions through multiple pathways, forming an interactive axis between the brain and intestines that exerts regulatory effects [10].

Studies have shown alterations in the abundance and types of bacteria in the intestines of MS patients, including changes in bacteria from Firmicutes and Bacteroidetes phyla [11]. Ochoa-Reparaz et al. [12] found a close relationship between butyrate and the differentiation of intestinal regulatory T cells (Tregs) as well as the integrity of the intestinal mucosal barrier. In MS patients, there is a significant decrease in the abundance of butyrate-producing bacteria in the intestines, indicating a dysregulation in the microbiota-gut-brain axis associated with MS and various diseases.

**2.2 Mechanisms of Gut Microbiota Influencing the Onset and Development of MS**

(1) Influence of Gut Microbiota on Th17/Treg Balance: In the host's immune regulatory system, CD4+ T lymphocytes, as targets of central nervous system myelin antigens, play an essential role together with other immune effectors in maintaining immune homeostasis and host defense, which is important in the pathogenesis of MS [13]. CD4+ T cells are a major cell population in the gut lymphocytes mediating host protection and homeostatic response [14]. Under the influence of gut microbiota, CD4+ T cells can differentiate into Tregs, Th17 cells, and other cell subgroups. Cytokines IL-17A, IL-17F, and IL-22 secreted by Th17 cells can induce the production of tight junction proteins and antimicrobial peptides by intestinal epithelial cells, participating in the occurrence of inflammation. The expression levels of transforming growth factor (TGF)-β influence T cell differentiation, with high TGF-β expression inducing the generation of adaptive regulatory T cells, while low expression favors Th17 differentiation [15]. Treg cells produce anti-inflammatory cytokines such as IL-10, IL-2, and IL-5, interacting with dendritic cell CD28 ligands, which are immunoprotective for the gut microbiota [16]. Studies have indicated that Bacteroides fragilis in the gut microbiota can stimulate the expression of Toll-like receptor 2 on CD4+ T cell surfaces via its capsule polysaccharide A, inducing the generation of Treg cells, thus promoting immune suppression beneficial for the gut and the body [17]. Researchers have identified Akkermansia muciniphila and Acinetobacter calcoaceticus as significantly associated bacteria in MS patients [18]. Numerous studies suggest that the composition of gut microbiota affects the balance of Treg/Th17 cells. The imbalance of Treg/Th17 cells is a major pathological factor in MS [19]. Therefore, the gut microbiota influences the onset of MS. In MS patients, there is a significant shift in Treg/Th17 cell differentiation, with an increased proportion of Th17 cells involved in inflammation and autoimmune diseases, while the proportion of Treg cells engaged in autoimmune tolerance regulation markedly decreases [20–21].

(2) Impact of Gut Microbiota on Blood-Brain Barrier Permeability: The blood-brain barrier is a complex cellular system located between brain capillary endothelial cells and neuroglial cells, maintaining the separation between blood plasma and brain cells. Branisle et al. [22] demonstrated that gut microbiota modulates blood-brain barrier (BBB) permeability by regulating tight junction protein expression in brain endothelial cells. Germ-free mice exhibited increased BBB permeability and reduced tight junction protein levels, which normalized after gut microbiota restoration. The BBB, a critical physiological barrier, prevents harmful substances from entering the brain, ensuring CNS stability. Increased BBB permeability allows immune cells and factors to infiltrate the CNS via the bloodstream, disrupting central immune responses and contributing to MS pathology [23]. Thus, gut microbiota may influence MS onset by altering BBB permeability.

(3) Influence of Gut Microbiota on Intestinal Barrier Permeability: The intestinal barrier comprises chemical, mechanical, immune, and biological barriers. Under normal conditions, these barriers collectively prevent harmful substances like pathogenic bacteria and toxins in the gut from crossing the intestinal mucosa into the host, maintaining internal environmental homeostasis to ensure normal physiological activities in the body. The intestinal mucosa-related tissues contain innate and adaptive immune cells, and these cells regulate their anti-inflammatory and pro-inflammatory responses by identifying microbial signals and specific antigens [24]. Studies have shown increased intestinal barrier permeability in relapsing-remitting MS patients [25]. Dysbiosis of the gut microbiota has been found to impair intestinal barrier functions in MS patients. In one study, abnormal expression of membrane-associated protein 1 in the intestines following Salmonella infection led to increased intestinal permeability [26]. However, limitations exist, including the small number of twins undergoing endoscopic sampling and interspecies differences in physiology and immune responses. Future studies should expand sample sizes to better clarify these bacteria's role in human MS. If the gut microbiota's role in MS is confirmed, new therapeutic approaches targeting microbial modulation may be developed. Current research remains preliminary, and future similar studies will provide more comprehensive insights into the relationship between MS and gut microbiota. When the intestinal barrier is compromised, various toxins and their metabolites can pass through the barrier, triggering immune response reactions in the body, which may induce the occurrence of MS and various diseases [FIG S1].


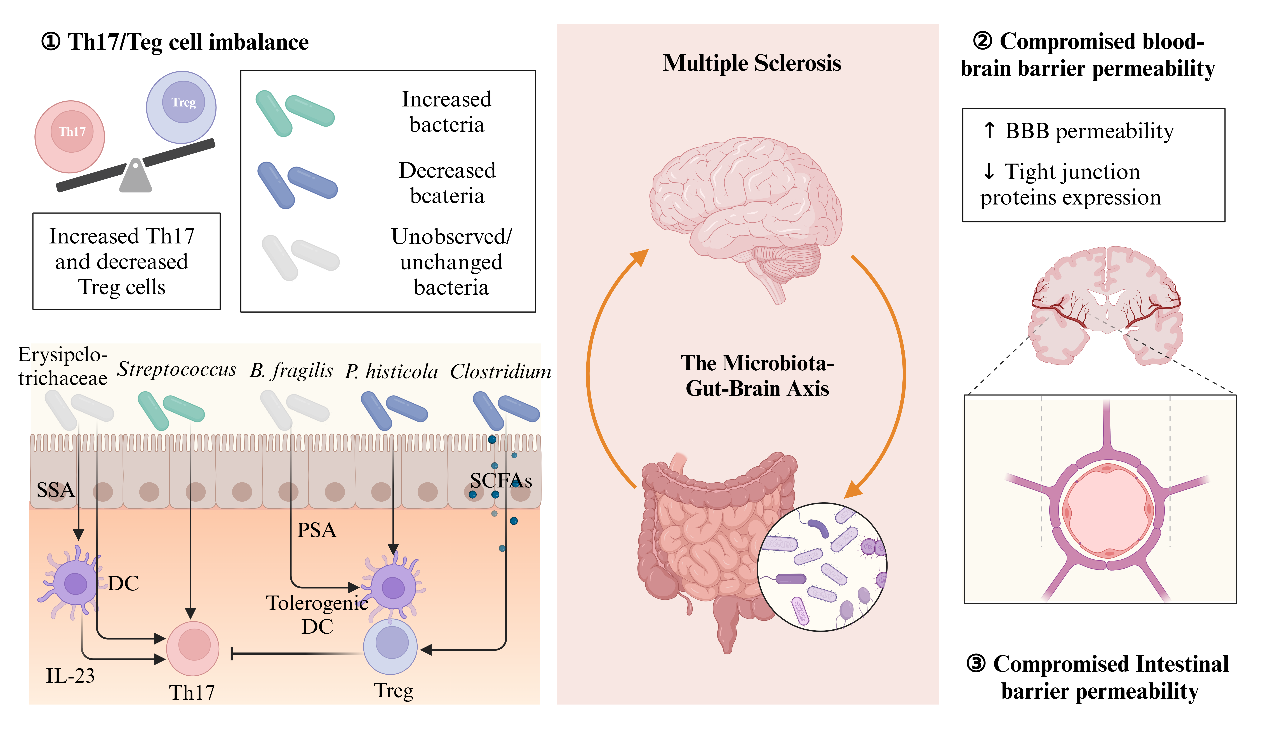


**FIG S1** Mechanisms of the gut microbiota in multiple sclerosis. The gut microbiota interacts with the central nervous system via the microbiota-gut-brain axis. In multiple sclerosis, dysbiosis disrupts the Th17/Treg balance. *Erysipelotrichaceae* family members stimulate serum amyloid A (SAA), activating dendritic cells and inducing IL-23 secretion, which promotes autoreactive Th17 cell expansion and CNS demyelination. Increased *Akkermansia muciniphila* and *Streptococcus* species in MS patients further drive Th17 differentiation. Conversely, *Bacteroides fragilis*, *Prevotella histicola*, and *Clostridium* species promote Treg cell generation through polysaccharide A (PSA) or short-chain fatty acids (SCFAs), potentially suppressing inflammation. Dysbiosis also disrupts tight junction proteins, compromising blood-brain and intestinal barrier permeability. This figure was created using BioRender.com.

**Abbreviations:** *B. fragilis*, *Bacteroides fragilis*; *P. histicola*, *Prevotella histicola*; SSA, serum amyloid A; DC, dendritic cell; PSA, polysaccharide A; SCFA, short-chain fatty acid; BBB, blood-brain barrier.

**2.3 Interventions of Gut Microbiota in the Prevention and Treatment of MS**

The abundance and species composition of gut microbiota influence the onset and development of MS. Probiotics, as a class of active microorganisms, act as "non-toxic immune modulators." The supplementation of probiotics can alter the composition and function of gut microbiota and, when used in conjunction with MS drugs, has the ability to enhance efficacy [27]. Studies have found that feeding a mixed preparation of L. plantarum A7 and B. animalis PTCC 1631 to experimental autoimmune encephalomyelitis (EAE) mice alleviates EAE symptoms. This effect is attributed to the probiotics' induction of anti-inflammatory factors (IL-4, IL-10, TGF-β) secretion and inhibition of pro-inflammatory factors (interferon, IL-17, IL-6, T-box transcription factor, vitamin D-related orphan receptor) [28]. Numerous studies demonstrate that probiotics play a positive role in improving central nervous system function and suppressing the onset and progression of MS [29–30]. Furthermore, research suggests that using fecal microbiota transplantation (FMT) to treat MS with severe constipation can improve intestinal function in patients [31]. Makkawi et al. [32] found that MS patients showed improved Expanded Disability Status Scale scores after receiving FMT treatment. FMT, as a microbial-targeted therapy, demonstrates relatively high safety, tolerance, and superiority, holding great potential for MS treatment. Reconstructing gut microbiota using the FMT method offers a new approach to treating diseases like MS [33–34]. Vitamin D is an essential micronutrient in the human body, playing a crucial role in regulating immune system function and preventing MS. Vitamin D deficiency can induce the production of forkhead/winged helix transcription factor 3 + Treg cells in the gut, leading to immune response alterations and gut microbiota disruption [35]. In a prospective study of over 18,700 healthy women, Munger et al. [36] proposed that vitamin D intake may modulate MS onset. Women with higher dietary vitamin D intake (approximately 700 IU/day) exhibited a lower risk of developing MS compared to those with lower intake [35]. Therefore, supplementing vitamin D plays a significant role in preventing and reversing MS, potentially due to its influence on gut microbiota. Studies indicate that rational use of antibiotics during the disease onset can regulate gut microbiota, which is crucial in delaying the progression of EAE [37]. Administering broad-spectrum antibiotics orally to mice can increase the number of regulatory CD5+ B lymphocytes, reducing susceptibility to EAE [37]. Additionally, moderate exercise, a balanced diet, and other factors can effectively maintain the stability of gut microbiota, exerting positive preventive and therapeutic effects on the onset and development of MS [38].

In summary, the gut microbiota regulates immunity, affects blood-brain barrier integrity, triggers autoimmune demyelination, and interacts with central nervous system cells. Cross-sectional studies show taxonomic changes in the gut microbiota of MS children compared to healthy controls, though α- or β-diversity differences are minimal. Research on microbiota transplantation into EAE models highlights CD4+ T cell-derived IL-10's role in immune regulation [39–41]. Segmented filamentous bacteria (SFB) may activate Th17 cells, influencing MS-like symptoms in EAE mice. Preclinical studies suggest gut microbiota regulates myelin production in mouse models, and GF mouse studies link microbial composition to blood-brain barrier disruption, a key feature of MS [40].

Meanwhile, dietary supplementation of SCFAs or SCFA-producing bacteria can restore blood-brain barrier integrity, and diet-induced gut microbiota changes influence EAE development [41]. The gut microbiota modulates neuroinflammatory pathways [42], but further research is needed to clarify its role in multiple sclerosis (MS) pathogenesis. Both animal and human studies suggest the gut microbiota impacts MS pathology, yet how to effectively manipulate it for disease prevention and treatment remains unclear. Pilot experiments show that specific probiotics (e.g., lactobacilli, bifidobacteria, streptococci) can reverse microbiota changes and regulate inflammation, highlighting potential for microbiota-targeted therapies. Further studies are required to validate these findings. In summary, given the extensive heterogeneity of MS and the tremendous complexity of symbiotic microbiota, future research directions clearly require larger-scale, more comprehensive third-generation microbiota studies to gain deeper insights into the role of microbiota in MS. These studies must be meticulously controlled to ensure more comprehensive findings. For instance, diet is the most significant factor influencing gut microbiota composition, so cases and control groups need to be balanced in terms of diet to ensure any differences in microbiota composition are disease-related rather than diet-related [43]. Beyond diet, gut microbiome composition is also affected by factors such as age, sex, ethnicity, geographic location, and environmental elements (e.g., smoking and exercise). The combination of these factors means associations between microbiota components and disease are often too weak to explain most of the variation observed in phenotypes. Large, multicenter controlled studies are necessary to minimize the impact of these factors and more definitively establish associations [Box S1].

| **Box S1 \| Gut Microbiota and Microbial Metabolites in Multiple Sclerosis Pathogenesis** |
| --- |
| **Short-chain Fatty Acids (SCFAs)**   - **Dysbiosis Feature:** Reduced butyrate-producing bacteria (*Blautia*, *Butyricoccaceae*) correlate with disease severity - **Mechanistic Role:** SCFAs maintain blood-brain barrier integrity via tight junction proteins; butyrate induces Treg differentiation - **Therapeutic Potential:** Dietary SCFA supplementation restores BBB function and suppresses EAE in mice   **Tryptophan Metabolites**   - **AhR Signaling:** Decreased serum AhR agonists (indole-3-aldehyde, IAA) in MS patients - **Microbial Link:***Bacteroides* spp. metabolize tryptophan to anti-inflammatory indoles - **Intervention Effect:** Probiotics increase fecal IAA, promoting Treg expansion and Th1 suppression via AhR activation   **Bile Acids**   - **Metabolic Shift:** Secondary bile acids (e.g., ursodeoxycholic acid) ↓ in progressive MS - **Neuroprotection:** TGR5 activation by bile acids reduces CNS inflammation in EAE models - **Biomarker Potential:** Serum palmitoleate↑ and UDCA↓ associate with MRI lesions   **Isoflavone-Metabolizing Bacteria**   - **Protective Taxa:***Paradiserella* and *Adlercreutzia* convert isoflavones to S-equol, attenuating EAE - **Diet-Microbe Interaction:** Isoflavone-rich diets reshape microbiota composition, reducing MOG-specific Th17 cells |

**References:**

1. Jakimovski D, Bittner S, Zivadinov R, Morrow SA, Benedict RH, Zipp F, Weinstock-Guttman B. Multiple sclerosis. Lancet. 2024;403(10422):183-202.doi: 10.1016/S0140-6736(23)01473-3.
2. Kujawa D, Laczmanski L, Budrewicz S, Pokryszko-Dragan A, Podbielska M. Targeting gut microbiota: new therapeutic opportunities in multiple sclerosis. Gut Microbes. 2023 ;15(2):2274126.doi: 10.1080/19490976.2023.2274126.
3. Altieri C, Speranza B, Corbo MR, Sinigaglia M, Bevilacqua A. Gut-Microbiota, and Multiple Sclerosis: Background, Evidence, and Perspectives. Nutrients. 2023;15(4):942.doi: 10.3390/nu15040942.
4. Bronzini M, Maglione A, Rosso R, Matta M, Masuzzo F, Rolla S, Clerico M. Feeding the gut microbiome: impact on multiple sclerosis. Front Immunol. 2023;14:1176016.doi: 10.3389/fimmu.2023.1176016.
5. iMSMS Consortium. Electronic address: [sergio.baranzini@ucsf.edu](mailto:sergio.baranzini@ucsf.edu); iMSMS Consortium. Gut microbiome of multiple sclerosis patients and paired household healthy controls reveal associations with disease risk and course. Cell. 2022;185(19):3467-3486.e16. doi: 10.1016/j.cell.2022.08.021.
6. Schwerdtfeger LA, Montini F, Lanser TB, Ekwudo MN, Zurawski J, Tauhid S, Glanz BI, Chu R, Bakshi R, Chitnis T, Cox LM, Weiner HL. Gut microbiota and metabolites are linked to disease progression in multiple sclerosis. Cell Rep Med. 2025 Apr 15;6(4):102055. doi: 10.1016/j.xcrm.2025.102055. Epub 2025 Apr 3. PMID: 40185103; PMCID: PMC12047500.
7. Peng HR, Qiu JQ, Zhou QM, Zhang YK, Chen QY, Yin YQ, Su W, Yu S, Wang YT, Cai Y, Gu MN, Zhang HH, Sun QQ, Hu G, Wu YW, Liu J, Chen S, Zhu ZJ, Song XY, Zhou JW. Intestinal epithelial dopamine receptor signaling drives sex-specific disease exacerbation in a mouse model of multiple sclerosis. Immunity. 2023;56(12):2773-2789.e8. doi: 10.1016/j.immuni.2023.10.016.
8. Collins SM, Surette M, Bercik P. The interplay between the intestinal microbiota and the brain. Nat Rev Microbiol. 2012 ;10(11):735-42. doi: 10.1038/nrmicro2876.
9. Schneider E, O'Riordan KJ, Clarke G, Cryan JF. Feeding gut microbes to nourish the brain: unravelling the diet-microbiota-gut-brain axis. Nat Metab. 2024;6(8):1454-1478. doi: 10.1038/s42255-024-01108-6.
10. Góralczyk-Bińkowska A, Szmajda-Krygier D, Kozłowska E. The Microbiota-Gut-Brain Axis in Psychiatric Disorders. Int J Mol Sci. 2022;23(19):11245. doi: 10.3390/ijms231911245.
11. Forbes JD, Vw Domselaw G, Bernstein CN. The gut microbiota in immune-mediated inflammatory diseases. Front Microbiol, 2016,7:1081.
12. Ochoa-Repáraz J, Kirby TO, Kasper LH. The Gut Microbiome and Multiple Sclerosis. *Cold Spring Harb Perspect Med*. 2018;8(6):a029017. Published 2018 Jun 1. doi:10.1101/cshperspect.a029017
13. Correale J, Hohlfeld R, Baranzini SE. The role of the gut microbiota in multiple sclerosis. Nat Rev Neurol. 2022 Sep;18(9):544-558. doi: 10.1038/s41582-022-00697-8.
14. Yoshimura A, Ohyagi M, Ito M. T cells in the brain inflammation. Adv Immunol. 2023;157:29-58. doi: 10.1016/bs.ai.2022.10.001.
15. Gomez-Bris R, Saez A, Herrero-Fernandez B, Rius C, Sanchez-Martinez H, Gonzalez-Granado JM. CD4 T-Cell Subsets and the Pathophysiology of Inflammatory Bowel Disease. Int J Mol Sci. 2023;24(3):2696. doi: 10.3390/ijms24032696.
16. Wang J, Zhao X, Wan YY. Intricacies of TGF-β signaling in Treg and Th17 cell biology. Cell Mol Immunol. 2023;20(9):1002-1022. doi: 10.1038/s41423-023-01036-7.
17. Round JL, Lee SM, Li J, Tran G, Jabri B, Chatila TA, Mazmanian SK. The Toll-like receptor 2 pathway establishes colonization by a commensal of the human microbiota. Science. 2011;332(6032):974-7. doi: 10.1126/science.1206095.
18. Cekanaviciute E, Yoo BB, Runia TF, Debelius JW, Singh S, Nelson CA, Kanner R, Bencosme Y, Lee YK, Hauser SL, Crabtree-Hartman E, Sand IK, Gacias M, Zhu Y, Casaccia P, Cree BAC, Knight R, Mazmanian SK, Baranzini SE. Gut bacteria from multiple sclerosis patients modulate human T cells and exacerbate symptoms in mouse models. Proc Natl Acad Sci U S A. 2017 Oct 3;114(40):10713-10718. doi: 10.1073/pnas.1711235114.
19. van Langelaar J, van der Vuurst de Vries RM, Janssen M, Wierenga-Wolf AF, Spilt IM, Siepman TA, Dankers W, Verjans GMGM, de Vries HE, Lubberts E, Hintzen RQ, van Luijn MM. T helper 17.1 cells associate with multiple sclerosis disease activity: perspectives for early intervention. Brain. 2018;141(5):1334-1349. doi: 10.1093/brain/awy069.
20. Wang HH, Dai YQ, Qiu W, Lu ZQ, Peng FH, Wang YG, Bao J, Li Y, Hu XQ. Interleukin-17-secreting T cells in neuromyelitis optica and multiple sclerosis during relapse. J Clin Neurosci. 2011 t;18(10):1313-7. doi: 10.1016/j.jocn.2011.01.031.
21. Li Y, Wang H, Long Y, Lu Z, Hu X. Increased memory Th17 cells in patients with neuromyelitis optica and multiple sclerosis. J Neuroimmunol. 2011 May;234(1-2):155-60. doi: 10.1016/j.jneuroim.2011.03.009. Epub 2011 Apr 12. PMID: 21489641.
22. Braniste V, Al-Asmakh M, Kowal C, Anuar F, Abbaspour A, Tóth M, Korecka A, Bakocevic N, Ng LG, Kundu P, Gulyás B, Halldin C, Hultenby K, Nilsson H, Hebert H, Volpe BT, Diamond B, Pettersson S. The gut microbiota influences blood-brain barrier permeability in mice. Sci Transl Med. 2014;6(263):263ra158. doi: 10.1126/scitranslmed.3009759.
23. Kujawa D, Laczmanski L, Budrewicz S, Pokryszko-Dragan A, Podbielska M. Targeting gut microbiota: new therapeutic opportunities in multiple sclerosis. Gut Microbes. 2023 Dec;15(2):2274126. doi: 10.1080/19490976.2023.2274126.
24. Leiner S, Vwderleyden J, Do Keersmaeckor SC. Host interoeons of probiotic bacterial surface molecules: comparison with commensals and pathogens. Nat Rev Micmbici, 2010, 8(3):171 -184.
25. Buscarinu MC, Cerasoli B, Annibali V, Policano C, Lionetto L, Capi M, Mechelli R, Romano S, Fornasiero A, Mattei G, Piras E, Angelini DF, Battistini L, Simmaco M, Umeton R, Salvetti M, Ristori G. Altered intestinal permeability in patients with relapsing-remitting multiple sclerosis: A pilot study. Mult Scler. 2017;23(3):442-446. doi: 10.1177/1352458516652498.
26. Spadoni I, Zagato E, Bertocchi A, Paolinelli R, Hot E, Di Sabatino A, Caprioli F, Bottiglieri L, Oldani A, Viale G, Penna G, Dejana E, Rescigno M. A gut-vascular barrier controls the systemic dissemination of bacteria. Science. 2015;350(6262):830-4. doi: 10.1126/science.aad0135. Yoon H, Gerdes LA, Beigel F, Sun Y, Kövilein J, Wang J, Kuhlmann T, Flierl-Hecht A, Haller D, Hohlfeld R, Baranzini SE, Wekerle H, Peters A. Multiple sclerosis and gut microbiota: Lachnospiraceae from the ileum of MS twins trigger MS-like disease in germfree transgenic mice-An unbiased functional study. Proc Natl Acad Sci U S A. 2025 May 6;122(18):e2419689122. doi: 10.1073/pnas.2419689122. Epub 2025 Apr 21. PMID: 40258140; PMCID: PMC12067282.
27. Kujawa D, Laczmanski L, Budrewicz S, Pokryszko-Dragan A, Podbielska M. Targeting gut microbiota: new therapeutic opportunities in multiple sclerosis. Gut Microbes. 2023;15(2):2274126. doi: 10.1080/19490976.2023.2274126.
28. Salehipour Z, Haghmorad D, Sankian M, et al. Bifidobacterium animalis in combination with human origin of Lactobacillus plantarum ameliorate neuroinflammation in experimental model of multiple sclerosis by altering CD4+ T cell subset balance. *Biomed Pharmacother*. 2017;95:1535-1548. doi:10.1016/j.biopha.2017.08.117
29. Asghari KM, Dolatkhah N, Ayromlou H, Mirnasiri F, Dadfar T, Hashemian M. The effect of probiotic supplementation on the clinical and para-clinical findings of multiple sclerosis: a randomized clinical trial. Sci Rep. 2023 Oct 30;13(1):18577. doi: 10.1038/s41598-023-46047-6.
30. Aburto MR, Cryan JF. Gastrointestinal and brain barriers: unlocking gates of communication across the microbiota-gut-brain axis. Nat Rev Gastroenterol Hepatol. 2024 Apr;21(4):222-247. doi: 10.1038/s41575-023-00890-0. Epub 2024 Feb 14. Erratum in: Nat Rev Gastroenterol Hepatol. 2024;21(5):365. doi: 10.1038/s41575-024-00929-w.
31. Yadegar A, Bar-Yoseph H, Monaghan TM, Pakpour S, Severino A, Kuijper EJ, Smits WK, Terveer EM, Neupane S, Nabavi-Rad A, Sadeghi J, Cammarota G, Ianiro G, Nap-Hill E, Leung D, Wong K, Kao D. Fecal microbiota transplantation: current challenges and future landscapes. Clin Microbiol Rev. 2024;37(2):e0006022. doi: 10.1128/cmr.00060-22.
32. Makkawi S, Camam-Remarmy C, Metz L. Fecal microbiota OansplantaWon associated with 10 years of stabDtp in a patient with SPMS. Neurol NeumiomunW NeuminUamm, 2018,5(4):e459.
33. Perler BK, Friedman ES, Wu GD. The Role of the Gut Microbiota in the Relationship Between Diet and Human Health. Annu Rev Physiol. 2023;85:449-468. doi: 10.1146/annurev-physiol-031522-092054.
34. Snigdha S, Ha K, Tsai P, Dinan TG, Bartos JD, Shahid M. Probiotics: Potential novel therapeutics for microbiota-gut-brain axis dysfunction across gender and lifespan. Pharmacol Ther. 2022 Mar;231:107978. doi: 10.1016/j.pharmthera.2021.107978.
35. Sangha A, Quon M, Pfeffer G, Orton SM. The Role of Vitamin D in Neuroprotection in Multiple Sclerosis: An Update. Nutrients. 2023;15(13):2978. doi: 10.3390/nu15132978.
36. Munger KL, Zhang SM, O'Reilly E, et al. Vitamin D intake and incidence of multiple sclerosis. *Neurology*. 2004;62(1):60-65. doi:10.1212/01.wnl.0000101723.79681.38
37. Shim JA, Ryu JH, Jo Y, Hong C. The role of gut microbiota in T cell immunity and immune mediated disorders. Int J Biol Sci. 2023;19(4):1178-1191. doi: 10.7150/ijbs.79430.
38. Brütting C, Stangl GI, Staege MS. Vitamin D, Epstein-Barr virus, and endogenous retroviruses in multiple sclerosis - facts and hypotheses. J Integr Neurosci. 2021;20(1):233-238. doi: 10.31083/j.jin.2021.01.392.
39. Noto D, Miyake S. Gut dysbiosis and multiple sclerosis. Clin Immunol. 2022 ;235:108380.doi: 10.1016/j.clim.2020.108380.
40. Ordoñez-Rodriguez A, Roman P, Rueda-Ruzafa L, Campos-Rios A, Cardona D. Changes in Gut Microbiota and Multiple Sclerosis: A Systematic Review. Int J Environ Res Public Health. 2023;20(5):4624.doi: 10.3390/ijerph20054624.
41. Samara A, Cantoni C, Piccio L, Cross AH, Chahin S. Obesity, gut microbiota, and multiple sclerosis: Unraveling the connection. Mult Scler Relat Disord. 2023 ;76:104768.doi: 10.1016/j.msard.2023.104768.
42. Dunalska A, Saramak K, Szejko N. The Role of Gut Microbiome in the Pathogenesis of Multiple Sclerosis and Related Disorders. Cells. 2023 Jun 30;12(13):1760.doi: 10.3390/cells12131760.
43. Correale J, Hohlfeld R, Baranzini SE. The role of the gut microbiota in multiple sclerosis. Nat Rev Neurol. 2022 Sep;18(9):544-558. doi: 10.1038/s41582-022-00697-8. Epub 2022 Aug 5. PMID: 35931825.
